# SINE‐Associated LncRNA *SAWPA* Regulates Porcine Zygotic Genome Activation

**DOI:** 10.1002/advs.202307505

**Published:** 2023-11-20

**Authors:** Tianyao He, Jinyu Peng, Shu Yang, Dongsong Liu, Shuang Gao, Yanlong Zhu, Zhuang Chai, Byeong Chun Lee, Renyue Wei, Jiaqiang Wang, Zhonghua Liu, Jun‐Xue Jin

**Affiliations:** ^1^ Key Laboratory of Animal Cellular and Genetics Engineering of Heilongjiang Province College of Life Science Northeast Agricultural University Harbin 150030 P. R. China; ^2^ Department of Theriogenology and Biotechnology College of Veterinary Medicine Seoul National University Seoul 08826 South Korea

**Keywords:** embryo development, lncRNA, short‐interspersed elements, transcriptional regulation, ZGA

## Abstract

In mice, retrotransposon‐associated long noncoding RNAs (lncRNA) play important regulatory roles in pre‐implantation development; however, it is largely unknown whether they function in the pre‐implantation development in pigs. The current study aims to screen for retrotransposon‐associated lncRNA in porcine early embryos and identifies a porcine 8‐cell embryo‐specific SINE‐associated nuclear long noncoding RNA named *SAWPA*. *SAWPA* is essential for porcine embryonic development as depletion of *SAWPA* results in a developmental arrest at the 8‐cell stage, accompanied by the inhibition of the JNK‐MAPK signaling pathway. Mechanistically, *SAWPA* works in *trans* as a transcription factor for *JNK* through the formation of an RNA‐protein complex with HNRNPA1 and MED8 binding the SINE elements upstream of *JNK*. Therefore, as the first functional SINE‐associated long noncoding RNAs in pigs, *SAWPA* provides novel insights for the mechanism research on retrotransposons in mammalian pre‐implantation development.

## Graphical abstract

During the early embryonic development of pigs, the SINE‐associated long non‐coding RNA *SAWPA* binds to MED8 and HNRNPA1 to regulate the transcription of *JNK* through SINE sequences, affecting the activation of the zygotic genome. Depletion of *SAWPA* leads to an impact on the JNK‐MAPK signaling pathway, causing embryonic development to become arrested at the 8‐cell stage.

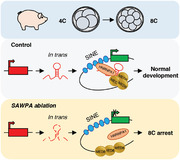

## 1 Introduction

Mechanism research on mammalian pre‐implantation development enhances our understanding of the origin and progression of our life. Emerging evidence indicates that long non‐coding RNAs (lncRNA) perform essential functions in gene regulation and epigenetic processes in mammalian pre‐implantation development. *XIST*‐mediated X chromosome inactivation,^[^ ^1^ ^]^ *Air* and *Kcnq1ot1*‐mediated gene imprinting,^[^ ^2^ ^]^ *pancIL17d*‐mediated DNA demethylation,^[^ ^3^ ^]^ and long intergenic non‐coding RNA *LincGET*‐mediated first cell fate bias^[^ ^4^ ^]^ are all essential for pre‐implantation development.

Transposable elements (TEs) are genetic elements in the genome that have the ability to move or transpose from one location to another. There are two main types of TEs: retrotransposons, which include short interspersed nuclear elements (SINEs), long interspersed nuclear elements (LINEs), and endogenous retroviruses (ERVs); and DNA transposons. They account for a substantial proportion of mammalian genomes (40% in mice and 44% in humans).^[^ ^5^ ^]^ TEs regulate gene transcription, chromatin structure and function, and cell differentiation.^[^ ^1^, ^6^ ^]^ TEs are major components of genomes, serving as main drivers of genome evolution while also being highly activated and transcribed during mammalian early embryonic development.^[^ ^7^ ^]^ In mammals, ≈80% of lncRNAs contain TEs, and these TEs‐associated lncRNAs play crucial roles in regulating embryonic development.^[^ ^8^ ^]^ The ERV‐associated lncRNA, *LincGET*, is essential for mouse embryonic development, and it plays an indispensable role in the determination of cell fate.^[^ ^4^, ^9^ ^]^ The SINE‐associated lncRNA, *Lx8‐SINE B2*, driven by Oct4 and Sox2, acts as a novel marker lncRNA by working with Eno1 in mouse ESCs.^[^ ^10^ ^]^ The activation of *LINE‐1* increases the global chromatin accessibility in early mouse embryos.^[^ ^11^ ^]^ The collective evidence indicates that TEs‐associated lncRNAs exert regulatory functions during the early stages of embryonic development.

The potential regulatory mechanisms with TEs‐associated lncRNA remain unclear. Interestingly, the totipotency pioneer factor, Nr5a2 activates ZGA by binding to the *SINE B1/Alu* transposable element in the *cis*‐regulatory element of the ZGA gene.^[^ ^12^ ^]^ Therefore, SINE‐mediated *cis*‐regulatory elements could have an important regulatory role in early embryonic development and merit further investigation. In addition, *cis*‐regulatory elements could mediate regulatory effects by transcribing lncRNAs. Some lncRNAs, such as *ncRNA‐a7* and *DXPas34*, serve as enhancer‐related lncRNAs,^[^ ^13^ ^]^ while the others, such as *ncRNA‐CCND1* and *iab‐7*,^[^ ^14^ ^]^ act as promoter‐related lncRNAs; some of them, such as *LINoCR* and *H19*, act as insulators.^[^ ^15^ ^]^ In addition, there are lncRNAs that could *trans*‐regulate gene transcription by binding *cis*‐regulatory elements containing repetitive sequences. *LincGET* achieves transcriptional regulation in *trans* by mediating the activity of the *cis*‐regulatory elements of GLK‐LTR.^[^ ^9^ ^]^ *HOTAIR* inhibits the expression of the Hox D gene cluster, which contains major regulatory genes involved in fore‐posterior axis formation, in *trans* by interacting with the PRC2 complex (catalyzes H3K27me3 establishment) and LSD1‐CoREST‐REST (catalyzes H3K4me2/3 erases).^[^ ^16^ ^]^ Therefore, SINE‐associated lncRNAs might regulate transcription both in *cis* and trans. This sheds new light on the mechanism underlying early embryonic development.

Research on SINE‐associated lncRNAs in early embryonic development has been relatively limited, and it remains unconfirmed whether these lncRNAs play a regulatory role in the early development of mammals. Here, we identified *SAWPA* (SINE‐associated and WDR26 promoter‐associated lncRNA) as a novel nuclear lncRNA. It is 8‐cell porcine embryo‐specific and is associated with SINEs. It is regulated by the partner mRNA, *WDR26*. *SAWPA* depletion leads to the developmental arrest at the 8‐cell stage and regulates porcine ZGA. It also decreases chromatin accessibility. *SAWPA* acts as a transcription factor via forming an RNA‐protein complex with HNRNPA1 and MED8; this mediates the *cis*‐regulatory activity of SINE and enhances the transcription of *JNK*. The inhibition of JNK phosphorylation or si_*JNK* injection decreased the pre‐implantation development and led to embryo arrest at the 8‐cell stage. To the best of our knowledge, this is the first study to demonstrate the essential role of SINE‐associated lncRNA in porcine ZGA processes. Additionally, we found that *SAWPA* promotes the cleavage of 8‐cell embryos by regulating the transcription of *JNK* through SINE elements. The discovery provides a novel regulatory mechanism for SINE‐associated lncRNAs that are involved in early embryonic development, shedding light on how lncRNAs are activated in ZGA during early embryonic development.

## 2 Result

### 2.1 SINE‐Associated LncRNAs Have High Expression During the ZGA Period in Pigs

**Figure 1:**
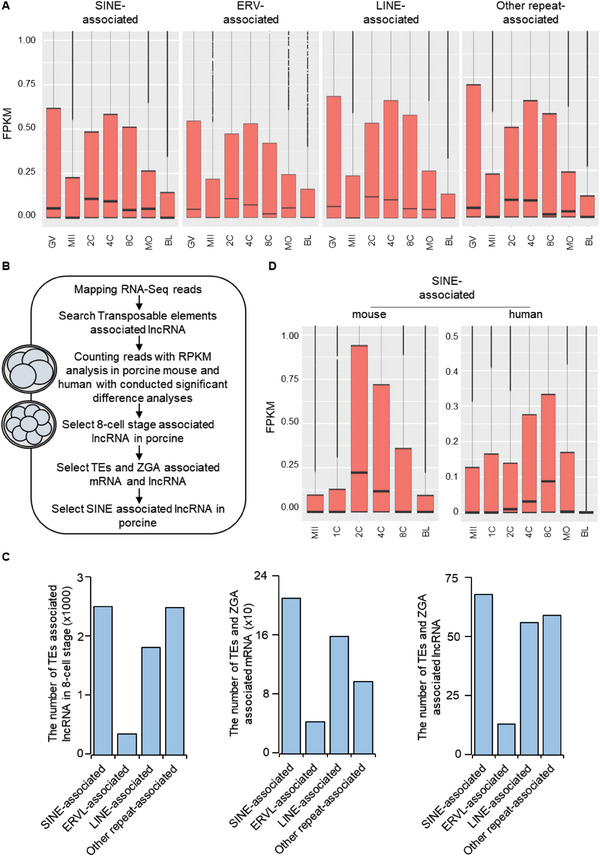
SINE‐associated lncRNA demonstrates elevated expression levels during the ZGA stage in pigs. A) The box plot for the expression levels analysis of TEs‐associated lncRNA during early embryonic development in porcine (GSE163620). GV, germinal vesicle oocyst; MII, metaphase of second meiosis; 2C, 2‐cell stage; 4C, 4‐cell stage; 8C, 8‐cell stage; MO, morula; BL, 7 days blastocyst. Significant difference analyses and *p*‐values can be found in Table S2 (Supporting Information). B) Screening of RNA seq datasets. C) The distribution of different types of TEs during the ZGA stage in porcine (GSE163620). D) Analysis of the expression levels of SINE‐associated lncRNA during early embryonic development in mice and humans (GSE138760, GSE36552). GV, germinal vesicle oocyst; MII, metaphase of second meiosis; 2C, 2‐cell stage; 4C, 4‐cell stage; 8C, 8‐cell stage; MO, morula; BL, 7 days blastocyst. Significant difference analyses and *p*‐values can be found in Table S2 (Supporting Information).

TEs‐associated lncRNAs play an active role in development.^[^ ^4^, ^17^ ^]^ To investigate the function of TEs‐associated lncRNAs in porcine pre‐implantation embryos, we analyzed early embryonic RNA‐seq data (Poly(A) selecting, rRNA depleting) to identify TEs‐associated lncRNAs. We classified TEs‐associated lncRNAs into SINE‐, LINE‐, ERV‐, and other repeat‐associated lncRNAs. These lncRNAs are crucial for the early development of mammalian embryos and are involved in processes such as ZGA, blastocyst formation rate, cell apoptosis, and pluripotency.^[^ ^18^ ^]^ Subsequently, We determine the type of lncRNA by identifying overlapping sequences between the exons of lncRNA and TEs. Considering the possible presence of more than one of TEs within a lncRNA. We conducted a statistical analysis for each type of TEs contained within the lncRNA, following the methods reported in published papers.^[^ ^19^ ^]^ By analyzing the RNA‐Seq data of pigs (GSE163620) and comparing the expression levels at various stages of pre‐implantation development, we observed that TEs‐associated lncRNAs exhibit higher expression levels during the 4–8‐cell stage (**Figure** **1**A,B), which is a critical period for ZGA in porcine.^[^ ^20^ ^]^ To validate this finding, we performed the same analysis on porcine RNA‐Seq data (CRA004237) once again and obtained consistent results (Figure S1A, Supporting Information). This implies that TEs‐associated lncRNAs may play a crucial role in ZGA in porcine.

To explore the function of TEs‐associated lncRNAs, we conducted an analysis of RNA‐Seq data from mice (GSE138760) and humans (GSE36552). We found that TEs‐associated lncRNAs also exhibit higher expression levels during the 2–4‐cell stage in mice and the 4–8‐cell stage in humans (representing the respective ZGA timepoints in mouse and human embryos^[^ ^21^ ^]^), indicating the conservation of TEs‐associated lncRNA function in ZGA across species (Figure S1A, Supporting Information). Furthermore, we performed a quantitative analysis of lncRNAs belonging to different TE types in the porcine RNA‐Seq data (GSE163620, CRA004237) at the 8‐cell stage. We found that SINE‐associated lncRNAs are mostly abundant during the 8‐cell stage (Figure 1C; Figure S1B, Supporting Information). Subsequently, we screened mRNA and lncRNA during ZGA in pigs. Based on previous research, we conducted differential gene analysis using DESeq2, where genes with a |log_2_ fold change| ≥ 1 at 8C/MII and a p adjust < 0.01 were identified as ZGA genes.^[^ ^22^ ^]^ When analyzing the number of different TE types in mRNA and lncRNA during the ZGA period, we found that SINE‐associated mRNA and lncRNA also exhibited the highest numbers (Figure 1C; Figure S1B, Supporting Information). Moreover, in the expression patterns of mice and humans, SINE‐associated lncRNA showed the highest expression levels during the ZGA period (Figure 1D), implying the significance of SINE‐associated lncRNAs during the ZGA period.

### 2.2 *SAWPA* Is a SINE‐Associated, Highly Expressed in 8‐Cell Stage, and Nuclear‐Localized LncRNA

**Figure 2:**
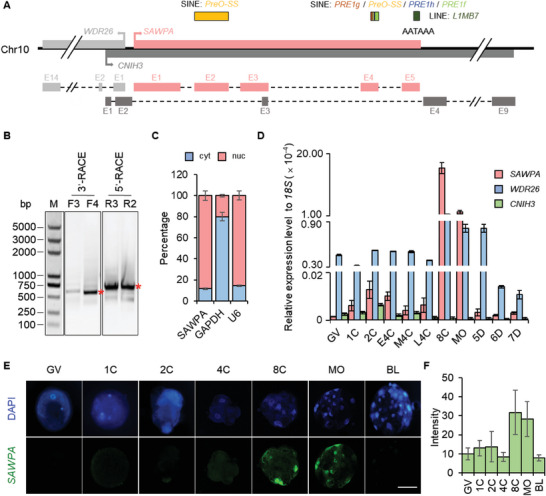
*SAWPA* is a SINE‐associated and nuclear‐located lncRNA. A) Gene locus of *SAWPA*. *SAWPA* is located upstream of *WDR26* and in the intron region of *CNIH3*. There are five SINE sequences and one LINE fragment of *L1MB7*. AATAAA is the polyadenylated signal site. B) 3′ RACE and 5′ RACE results for *SAWPA*. Gene‐specific primers (F3, F4, R3, and R2) are shown in Figure S1A (Supporting Information). *Indicates the bands corresponding to the correct band of 3′ RACE and 5′ RACE for *SAWPA*. Approximately 200 early 8‐cell embryos were used for each RACE experiment, and three experimental replicates were used. C) Subcellular localization of *SAWPA* using RNA fractionation and qPCR analysis. *SAWPA* is localized in the nucleus. The error bars represent S.E.M. Nuc, nucleoplasm; Cyt, cytoplasm. *GAPDH* and *U6* act as Cyt and Nuc control, respectively. About 200 early 8‐cell embryos were used for each experiment, and three experimental replicates were used. D) Expression pattern of *SAWPA*, *WDR26*, and *CNIH3* at different stages of preimplantation porcine embryos analyzed using qPCR. GV, germinal vesicle oocyst; 1C, 1‐cell stage; 2C, 2‐cell stage; E4C, early 4‐cell stage; M4C, middle 4‐cell stage; L4C, late 4‐cell stage; 8C, 8‐cell stage; MO, morula; 5D, 5 days blastocyst. 6D, 6 days blastocyst; 7D, 7 days blastocyst. The error bars represent S.E.M. Approximately 50 embryos of each stage were used, and three experimental replicates were used. E) RNA‐FISH in the oocyte to blastocyst embryos for *SAWPA*. *SAWPA* is present in the nucleus of 8‐ to morula‐cell embryos. GV, germinal vesicle oocyst; 1C, 1‐cell stage (*n* = 10 for each probe); 2C, 2‐cell stage (*n* = 8 for each probe); 4C, 4‐cell stage (*n* = 9 for each probe); 8C, 8‐cell stage (*n* = 11 for each probe); MO, morula (*n* = 10 for each probe); BL, blastocyst (*n* = 10 for each probe). Scale bar, 50 µm. Three experimental replicates were used. F) Fluorescence intensity analysis showed that *SAWPA* was expressed specifically in the 8‐cell and morula stages. Three experimental replicates were performed, and ≈20 embryos were used in each group.

In order to investigate the role of SINE‐associated lncRNA in ZGA, we specifically selected newly discovered transcripts that were upregulated during the 4–8‐cell stage and maintained high levels of expression. By sorting these transcripts based on their differential expression levels between the 4‐cell and 8‐cell stages, we systematically selected lncRNAs with significant expression changes for interference experiments. Subsequently, we closely monitored the embryonic development process. Through this methodical process, we ultimately identified *SAWPA*, named based on its functional attributes and positional characteristics. Using the UCSC blat tool (http://genome.ucsc.edu/cgi‐bin/hgBlat), we found *SAWPA* is located on chromosome 10 in the opposite direction of transcription of the *WDR26* gene. It is a transcript of an intron located in the *CNIH3* gene (**Figure** **2**A). Due to the sequencing data analyzed lacking complete information for the full length, we performed 3′ RACE and 5′ RACE to obtain the full‐length *SAWPA*. There were no other variants of *SAWPA*; it was a 1380 bp transcript consisting of 5 exons (Distinguished by the AT‐GC regio). Exon 2 contains the SINE element, Pre0‐SS; exon 4 contains 4 SINE elements, *PRE1g*, *Pre0‐SS*, *PRE1h*, and *PRE1*; and exon 5 contains the Line element, *L1MB7* (Figure 2A,B; Figure S2A, Supporting Information).

We assessed whether *SAWPA* are lncRNAs. Initially, we used NCBI ORF Finder (http://www.ncbi.nlm.nih.gov/projects/gorf/) to analyze the open‐reading frames (ORFs) of *SAWPA* and assess its coding potential. The results revealed several short ORFs. A subsequent comparison of these regions using the NCBI BLAST tool (NCBI/BLAST/blastp suite) failed to identify any conserved protein domains or kozak sequences. Therefore, *SAWPA* likely does not have coding potential (Figure S2B,C, Supporting Information). To further confirm this, we analyzed the subcellular localization of *SAWPA* using RNA extraction and SYBR Green real‐time quantitative PCR (qPCR) assay. We divided 8‐cell stage porcine embryos into two fractions: cytoplasmic (Cyt) and nuclear‐soluble (Nuc), and examined the expression of *SAWPA* in each fraction; GAPDH and U6 were used as controls. *SAWPA* was predominantly localized in the nucleus, further supporting its non‐coding status as lncRNA (Figure 2C).

We performed qPCR analysis of *SAWPA* expression at various embryonic development stages. The expression of *SAWPA* was relatively constant and below the detectable range during the GV stage oocyte to 4‐cell stage embryo. However, it dramatically increased and peaked at the 8‐cell stage; it then decreased to below the detectable range level in blastocysts (Figure 2D). We analyzed *SAWPA* expression in ESCs and various tissues; the expression was the highest in the 8‐cell stage (Figure S2D, Supporting Information). RNA‐FISH revealed high *SAWPA* levels in the nuclei of the 8‐cell and morula stages (Figure 2E). The fluorescence intensity test also proved that *SAWPA* expression was the highest at the 8‐cell stage (Figure 2F). Therefore, *SAWPA* is an 8‐cell stage embryo‐specific SINE‐associated nuclear lncRNA during preimplantation.

### 2.3 Interference with *SAWPA* Results in Embryo Arrest at 8‐Cell Stage and Affects the Activation of the Zygote Genome

**Figure 3:**
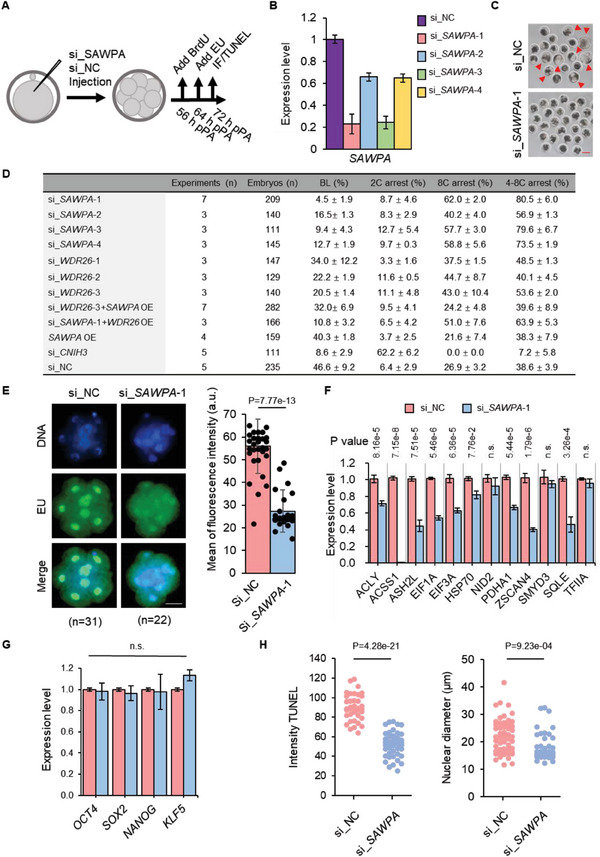
*SAWPA* depletion results in developmental arrest at the 8‐cell stage with effects on ZGA initiation. A) Experimental scheme to analyze the effects of *SAWPA* depletion on embryonic development. pPA, post‐parthenogenetic activation; IF, immunofluorescence. For BrdU staining, BrdU was added at pPA 56 h. For EU staining, EU was added at pPA 64 h. IF, including detection of BrdU and EU, was performed at pPA 72 h. B) RNAi efficiently mediated *SAWPA* knockdown. si_*SAWPA*‐1 was injected at pPA 6 h, and embryos were collected at pPA 72 h at the 8‐cell stage for qPCR analysis. The error bars represent S.E.M. Approximately 50 embryos of each stage were used, and three experimental replicates were used. *18S* is an internal reference gene. C) si_*SAWPA*‐1 embryos arrest at 8‐cell stage. The photographs were taken at pPA 168 h at the blastocyst stage. Embryos injected with si_NC can develop to the blastocyst stage, while si_*SAWPA*‐1 embryos are arrested at the 8‐cell stage. Scale bar, 100 µm. At least three experimental replicates were performed for each RNAi injection. D) Embryonic development after microinjection. 8C, 8‐cell stage; 4–8C, 4‐ to 8‐cell stage; BL, blastocyst stage; si_, siRNA; OE, overexpression. Differences of data [mean ± standard error of the mean (s.e.m.)] were analyzed by using a two‐tailed Student's *t*‐test. Specific *p*‐values can be found in Table S3 (Supporting Information). E) EU staining indicates the abnormal major ZGA process following si_*SAWPA*‐1 injected at the 1‐cell stage. EU was added to the culture medium at pPA 64 h, and EU signals were detected at pPA 72 h. The si_NC fluorescence intensity was significantly higher than that of si_*SAWPA*‐1. Scale bar, 50 µm. Three experimental replicates were used. The error bars represent S.E.M. F) Expression of genes related to major ZGA initiation, such as *ACLY*, *ACSS1*, *ASH2L*, *EIF1A*, *EIF3A*, *HSP70*, *NID2*, *PDHA1*, *ZSCAN4*, *SMYD3*, *SQLE*, and *TFIIA* in comparison between si_*SAWPA*‐1 and si_NC embryos. Embryos injected with RNAi were collected at pPA 72 h at the 8‐cell stage for qPCR analysis. The error bars represent S.E.M. Approximately 100 embryos were used for each group, and three experimental replicates were used. n.s., *p* > 0.05. *18S* is an internal reference gene. G) Expression of genes related to pluripotency, such as *OCT4*, *SOX2*, *NANOG*, and *KLF4*, are expressed normally in si_*SAWPA*‐1 embryos compared to that in si_NC embryos. Embryos injected with RNAi were collected at pPA 72 h at the 8‐cell stage for qPCR analysis. The error bars represent S.E.M. Approximately 100 embryos were used for each group, and three experimental replicates were used. n.s., P >0.05. H) *SAWPA* depletion decreased the fluorescence intensity of the TUNEL assay and nuclear volume in the 8‐cell stage. Two‐tailed Student's *t*‐tests were used for statistical analysis. The number of si_NC and si_*SAWPA*‐1 were 19 and 27. Three experimental replicates were used.

To explore the function of *SAWPA* in porcine embryonic development, an RNA interference assay (RNAi) was performed. Embryos were microinjected with negative control siRNA (si_NC) or siRNA targeting *SAWPA* (si_*SAWPA*) at the pronuclear stage (**Figure** **3**A). We screened four interference fragments and found that si_*SAWPA*‐1 and si_*SAWPA*‐3 achieved nearly 80% interference efficiency (Figure 3B). We then evaluated embryonic development and observed that depletion of *SAWPA* (si_*SAWPA*‐1) led to developmental arrest at the 8‐cell stage in porcine embryos (Figure 3C). We assessed the embryonic development following the injection of the relevant RNA fragments (Figure 3D). Our findings revealed a clear arrest in development at the 8‐cell stage for embryos injected with si_*SAWPA*‐1 and si_*SAWPA*‐3, with si_*SAWPA*‐1 exhibiting the most pronounced inhibitory effect. Consequently, we selected si_*SAWPA*‐1 as the interference fragment for subsequent studies. In addition, si_*SAWPA*‐2 and si_*SAWP*‐4 did not cause an obvious interference with the expression of *SAWPA* (but they did reduce the expression level of *SAWPA* by ≈30%); however, embryonic development was still arrested at the 8‐cell stage. At the same time, we found that overexpression of *SAWPA* did not affect embryonic development. This indicated that *SAWPA* could play an important role in the development of zygotic genome activation (ZGA) in porcine embryos. ZGA occurs in porcine between the 4‐cell and 8‐cell stages.^[^ ^20^ ^]^ The expression of *SAWPA* during the early, middle, and late 4‐cell embryonic stages was below the detection limit; therefore, we focused our follow‐up experiments on the 8‐cell stage arrest.

To investigate the impact of *SAWPA* interference on ZGA, we performed immunofluorescence staining (IF) for BrdU (added at post‐PA 56 h) and visualized DNA replication. *SAWPA*‐depleted 8‐cell embryos (si_*SAWPA*‐1 8‐cell) exhibited an interphase chromatin status and strong BrdU staining (Figure S3A, Supporting Information). To assess the initiation of major ZGA in *SAWPA*‐depleted 8‐cell embryos, we used 5′‐ethynyluridine (EU) staining (added at post‐PA 64 h) to detect total *de novo* transcripts (test at 72 h). si_NC and si_*SAWPA*‐1 were injected at the 1‐cell stage. The 8‐cell stage embryo of si_NC and si_*SAWPA*‐1 showed significant differences in EU signals, with the fluorescence intensity of si_*SAWPA*‐1 8‐cell being lower than si_NC (Figure 3E). These findings suggest that transcription was affected by *SAWPA* interference in the 8‐cell stage embryos.

ZGA is a crucial process in the maternal‐to‐embryonic transition and the establishment of the totipotent state.^[^ ^23^ ^]^ Various genes such as *ACLY*, *ACSS1*, *ASH2L*, *EIF1A*, *EIF3A*, *HSP70*, *NID2*, *PDHA1*, *ZSCAN4*, *SMYD3*, *SQLE*, and *TFIIA* are actively transcribed during ZGA.^[^ ^24^ ^]^ To investigate the effect of *SAWPA* interference on ZGA, we analyzed the expression levels of ZGA initiation genes using qPCR. Compared with si_NC, the expression of ZGA‐associated genes *ACLY*, *ACSS1*, *ASH2L*, *EIF1A*, *EIF3A*, *PDHA1*, *SMYD3*, *SQLE*, and *ZSCAN4* was decreased in si_*SAWPA*‐1 (Figure 3F). We evaluated the expression of genes associated with pluripotency, such as *OCT4*, *SOX2*, *NANOG*, and *KLF4*, which are transcription factors that regulate ZGA. However, there was no significant difference between si_*SAWPA*‐1 and si_NC (Figure 3G). Thus, *SAWPA* depletion has a significant effect on the initiation of ZGA by specifically influencing gene expression.

Global chromatin accessibility is an important factor that influences ZGA.^[^ ^25^ ^]^ To investigate whether si_*SAWPA*‐1 affects the activation of ZGA by influencing chromatin openness, we performed an *in* *vivo* DNase I‐TUNEL assay to detect chromatin openness. si_*SAWPA*‐1 led to significantly lower levels of TUNEL staining compared to that in the si_NC control blastomeres (Figure 3H; Figure S3B,C, Supporting Information). We compared nuclear volumes, which is another parameter for chromatin openness.^[^ ^11^ ^]^ There was a significant decrease in nuclear volume after si_*SAWPA*‐1 compared to that in the si_NC control blastomeres (Figure 3H; Figure S3C, Supporting Information). This indicates that si_*SAWPA*‐1 in 8‐cell embryos affects global chromatin accessibility.

### 2.4 *SAWPA* Depletion Does Not Affect the Expression of the Neighbor Gene

**Figure 4:**
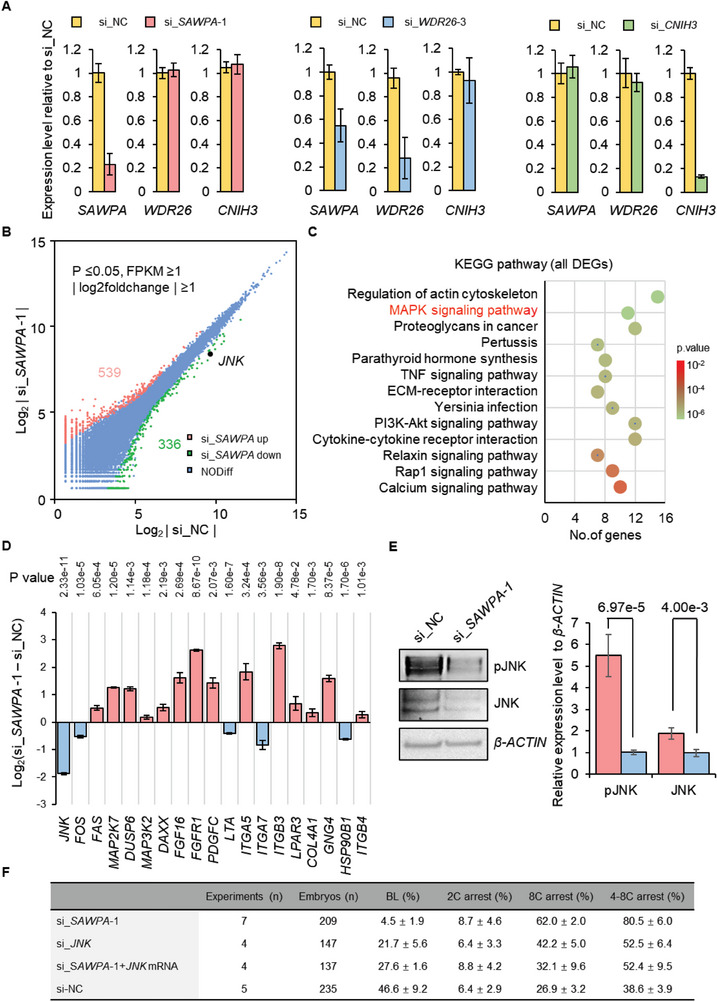
*SAWPA* depletion results in the inhibition of the MAPK signaling pathway and downregulates the expression of the SINE‐associated gene *JNK*. A) *SAWPA*, *WDR26*, and *CNIH3* expression analysis relative to the control group after RNAi with si_NC and negative control siRNA; si_*SAWPA*‐1, RNAi of *SAWPA*; *si_WDR26‐3*, RNAi of *WDR26*; si_*CNIH3*, RNAi of *CNIH3*; The error bars represent S.E.M. About 50 embryos of each stage were used, and three experimental replicates were used. 18S is an internal reference gene. B) DEGs analysis based on RNA‐seq data. Compared to that in the si_NC 8‐cells, 539 genes were upregulated and 226 genes were downregulated in si_*SAWPA*‐1 embryos. Approximately 400 embryos were used for each group, and two experimental replicates were used. C) KEGG pathway analysis of DEGs showed that the MAPK and PI3K‐Akt signaling pathways are mainly affected by *SAWPA* depletion. D) The expression of genes related to the JNK‐MAPK or PI3K‐Akt signaling pathways was significantly affected by *SAWPA* depletion. Embryos injected with si_*SAWPA*‐1 were collected at pPA 72 h at the 8‐cell stage for qPCR analysis. The error bars represent S.E.M. Approximately 80 embryos were used for each group, and three experimental replicates were used. Two‐tailed Student's *t*‐test was used for statistical analysis. E) Western blot and grayscale analysis indicate that the protein and phosphorylation levels of JNK and pJNK, key kinases in the MAPK signaling pathway, decreased in *SAWPA*‐depleted 8‐cell. Embryos injected with si_*SAWPA*‐1 and si_NC were collected at pPA 72 h at the 8‐cell stage for western blot analysis, and ≈200 embryos were used for each lane. The error bars represent S.E.M. Three experimental replicates were used. F) Embryonic development after si_*SAWPA*‐1 and si_*JNK* microinjection. 8C, 8‐cell stage; 4–8C, 4‐ to 8‐cell stage; BL, blastocyst stage; si_, siRNA. Differences of data [mean ± standard error of the mean (s.e.m.)] were analyzed by using a two‐tailed Student's *t*‐test. Specific *p*‐values can be found in Table S4 (Supporting Information).

To determine whether there is a regulatory relationship between *SAWPA* and the mRNAs of its neighboring genes, we analyzed the expression patterns of *SAWPA*, *WDR26*, and *CNIH3* at different stages of porcine preimplantation embryos using qPCR. *WDR26* had an expression pattern similar to that of *SAWPA*, with higher expression in the 8‐cell stage. *CNIH3* was detected only in the 2‐cell stage (Figure 2D). We performed RNA interference of *SAWPA*, *WDR26*, and *CNIH3* on 1‐cell stage embryos; qPCR analysis showed that RNAi of *SAWPA* and CNIH3 did not affect the expression of *WDR26*. However, RNAi of *WDR26* significantly decreased the expression of *SAWPA* (**Figure** **4**A). These results increased the possibility of *WDR26* regulating *SAWPA* expression.

We conducted a developmental analysis of embryos following RNAi of WDR26 and CNIH3. RNAi of WDR26 influenced embryonic development, with some embryos arrested in the 8‐cell stage. Interference with *CNIH3* blocked embryonic development at the 2‐cell stage (Figure 3D). Therefore, *SAWPA* could have a regulatory relationship with *WDR26* but not with *CNIH3*. We further investigated this by co‐injecting full‐length *SAWPA* with *si_WDR26‐*3; this partially rescued embryonic development to the 8‐cell stage. However, co‐injecting full‐length *WDR26* with si_*SAWPA*‐1 had no effect on rescuing embryonic development (Figure 3D; Figure S4A,B, Supporting Information). To explore the relationship between *SAWPA* and *WDR26*, we performed a double luciferase reporter system experiment; *WDR26* mRNA can enhance the fluorescence of *SAWPA* promoter‐pGL3‐luciferase vectors after interfering with endogenous *WDR26* (Figure S4C, Supporting Information). In summary, we confirmed that *WDR26* is indeed an upstream regulatory factor of *SAWPA*.

### 2.5 *SAWPA* Depletion Leads to JNK Signaling Pathway Inhibition

To investigate the mechanism of porcine embryonic development arrest at the 8‐cell stage after interfering with *SAWPA*, we collected 1600 8‐cell stage embryos injected with si_NC and si_*SAWPA*‐1 at 1‐cell stage, respectively, and subjected them to low sample volume RNA‐Seq. Compared to si_NC, si_*SAWPA*‐1 resulted in the deregulation of 875 genes, including 539 upregulated and 336 downregulated genes, referred to as differentially expressed genes (DEGs) (*P* ≤  0.05, FPKM ≥ 1, and |log_2_foldchange| ≥ 1) (Figure 4B). KEGG pathway analysis of DEGs suggested that *SAWPA* depletion disrupted the MAPK signaling pathway by inhibiting key factors in the JNK‐MAPK signaling pathways (Figure 4C; Figure S5A, Supporting Information). In addition, *SAWPA* depletion affected the PI3K‐AKT signaling pathway but did not affect the key factors in the pathway. We verified the key factors responsible for changes in expression in the MAPK and PI3K‐AKT signaling pathways using qPCR. The expression of *JNK* in *SAWPA*‐depleted embryos decreased by approximately four times (Figure 4D), and western blotting indicated a decrease in JNK phosphorylation levels and a decrease in total *JNK* protein levels in *SAWPA*‐depleted embryos (Figure 4E). The results indicate that the JNK signaling pathway plays a vital role in early embryonic development in porcine.

To verify whether the JNK signaling pathway is a key signaling pathway for ZGA in porcine, we analyzed the expression level of *JNK* in various stages of embryonic development using qPCR. The expression of *JNK* was high during the embryonic development from the 4‐cell to 8‐cell stage, suggesting that *JNK* played an important role in the ZGA during the early embryonic development in pigs (Figure S5B, Supporting Information). We injected si_*JNK* and si_*SAWPA*‐1 at the 1‐cell stage and detected the expression levels of *JNK* and *SAWPA* using qPCR. *SAWPA* depletion affected the expression level of *JNK*, while *JNK* depletion did not affect the expression level of *SAWPA*, implying that there is a regulatory relationship between *SAWPA* and *JNK* (Figure 4D; Figure S5C, Supporting Information). The embryos injected with si_*JNK* were affected by blastocyst rate, and embryos arrest and 8‐cell stage. We then co‐injected si_*SAWPA*‐1 embryos with overexpression of *JNK* mRNA; overexpressed *JNK* rescued embryonic development arrest compared to the si_*SAWPA*‐1 group (Figure 4F).

To evaluate whether *SAWPA* depletion at the 8‐cell stage affects development by influencing JNK protein phosphorylation levels, we analyzed the expression level of pJNK in various stages of embryonic development through western blotting. The expression of pJNK was high during embryonic development from the 4‐cell to 8‐cell stage (Figure S5D, Supporting Information). We then statistically analyzed embryonic development by interfering with JNK and treatment with pJNK inhibitors (TCS JNK 6o). Interference with JNK‐MAPK led to a significant decrease in the embryonic development blastocyst rate (**Table** **1**). Some embryos were arrested in the 8‐cell stage. When different concentrations of the JNK inhibitor, TCS *JNK* 6o, were added, the 1 and 5 µm treatments had little effect on development. However, the blastocyst rate of embryos in the 10, 20, 25, 50, and 100 µm groups decreased significantly, thereby impeding embryonic development (Table 1). Western blotting for pJNK in embryos treated with inhibitors revealed that the low concentrations of 1 and 5 µm could not inhibit the phosphorylation of *JNK* in the 8‐cell stage (Figure S5E, Supporting Information). In order to make the results more accurate, we conducted gray value analysis on the western blotting results and found that pJNK had a high expression level at the 8‐cell stage, and JNK phosphorylation screen breakage was successfully inhibited by the inhibitor, which verified the previous results This finding further confirms the significance of JNK phosphorylation during the ZGA period (Figure S5F,G, Supporting Information). In conclusion, the depletion of *SAWPA* led to the arrest of embryonic development at the 8‐cell stage by influencing the JNK signaling pathway.

**Table 1:** Embryonic development after microinjection or inhibition treatment.

	Experiments [n]	Embryos [n]	BL [%]	2C arrest [%]	8C arrest [%]	4–8C arrest [%]
DMSO	4	221	43.6 ± 5.8	4.9 ± 3.0	19.1 ± 4.4	24.5 ± 6.9
1 µm TCS JNK 6o	4	308	44.1 ± 4.7	5.3 ± 2.3	20.8 ± 5.2	30.3 ± 7.4
5 µm TCS JNK 6o	4	329	41.4 ± 7.2	5.9 ± 4.0	22.2 ± 7.9	30.4 ± 9.3
10 µm TCS JNK 6o	4	306	27.2 ± 7.1	6.3 ± 1.7	39.4 ± 9.4	46.0 ± 10.5
20 µm TCS JNK 6o	4	329	25.9 ± 8.0	9.2 ± 4.9	39.3 ± 6.7	46.8 ± 8.3
25 µm TCS JNK 6o	3	106	22.7 ± 3.8	6.5 ± 3.8	39.4 ± 5.6	48.9 ± 5.4
50 µm TCS JNK 6o	3	85	15.4 ± 2.4	12.6 ± 8.2	38.7 ± 3.1	46.8 ± 8.5
100 µm TCS JNK 6o	3	95	10.3 ± 8.5	7.9 ± 6.4	58.9 ± 0.3	66.3 ± 1.5

8C, 8‐cell stage; 4–8C, 4‐ to 8‐cell stage; BL, blastocyst stag. Differences of data [mean ± standard error of the mean (s.e.m.)] were analyzed by using a two‐tailed Student's *t*‐test. Specific *p*‐values can be found in Table S5 (Supporting Information).

### 2.6 *SAWPA* Binds to HHNRNPA1 and MED8

**Figure 5:**
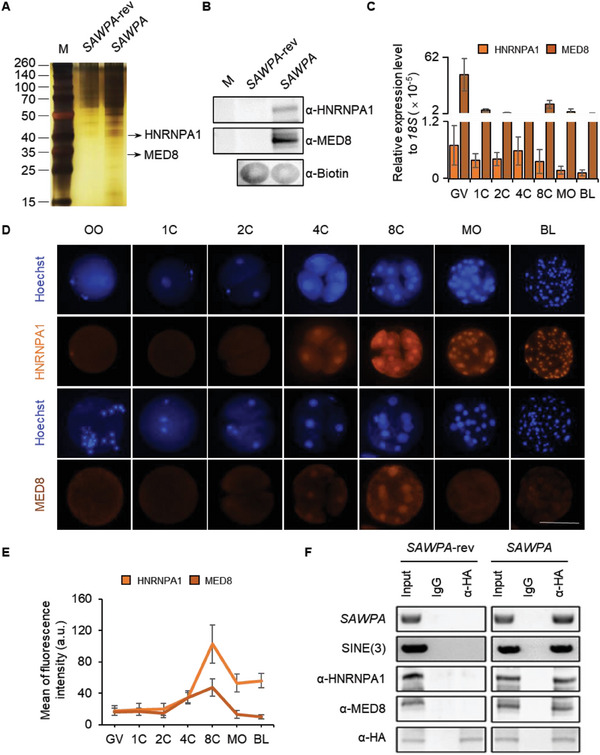
*SAWPA* binds to HNRNPA1 and MED8. A) *SAWPA* interacts with HNRNPA1 and MED8 *in* *vitro*. The samples of RNA pull‐down experiment were subjected to SDS‐PAGE gel electrophoresis, and then the binding protein of *SAWPA* and *SAWPA*‐rev was analyzed by silver staining and MS. Only one pull‐down assay for mass spectrometry analysis was performed with 2000 early 8‐cell stage embryos. Two specific bands in the right lane (arrow) were analyzed through mass spectrometry and confirmed as HNRNPA1 and MED8. B) Mass spectrometry results of HNRNPA1 and MED8 were confirmed by Western blot following RNA pull‐down assay (pull‐down WB); *SAWPA*‐rev, reverse sequence of *SAWPA*. α‐, anti‐. For each pull‐down WB assay, ≈500 eight‐cell stage embryos were used and three experimental replicates were performed. C) Expression pattern of HNRNPA1 and MED8 at different stages of preimplantation porcine embryos analyzed using qPCR. GV, germinal vesicle oocyst; 1C, 1‐cell stage; 2C, 2‐cell stage; 4C, 4‐cell stage; 8C, 8‐cell stage; MO, morula; BL, 7 days blastocyst. The error bars represent S.E.M. Approximately 50 embryos of each stage were used, and three experimental replicates were used. D) IF assay in the oocyte to blastocyst embryos for HNRNPA1 and MED8. HNRNPA1 is present in the nucleus of 4‐cell to blastocyst‐cell embryos. MED8 is present in the nucleus of 4‐ cell to 8‐cell embryos GV, germinal vesicle oocyst (*n* = 19, 13); 1C, 1‐cell stage (*n* = 17, 14); 2C, 2‐cell stage (*n* = 22, 21); 4C, 4‐cell stage (*n* = 33, 17); 8C, 8‐cell stage (*n* = 42, 23); MO, morula (*n* = 48, 34); BL, blastocyst (*n* = 37, 29). Scale bar, 100 µm. Three experimental replicates were used. E) Fluorescence intensity analysis showed that HNRNPA1 and MED8 were highest expressed at 8‐cell stage embryos. Three experimental replicates were performed. F) Co‐IP results in eight‐cell stage embryos (*SAWPA‐*MS2 was injected at the 1‐cell stage) using anti‐HA (for HA‐labeled MS2 coat protein). The results show that *SAWPA* forms an RNA‐protein complex with HNRNPA1 and MED8. *SAWPA*‐rev, antisense sequence of *SAWPA* overexpression; *SAWPA*, *SAWPA* overexpression; The primer of RT‐PCR *SAWPA*‐1 were used to test *SAWPA*; SINE(3), DNA sequence of 3 (SINE element of *SAWPA*); For each co‐IP assay, ≈500 eight‐cell embryos were used, and three experimental replicates were performed.

To investigate the mechanism that *SAWPA* functions, we performed RNA pull‐down‐mass spectrometry with biotin‐labeled *SAWPA* to elucidate the proteins that interact with *SAWPA* using 2000 8‐cell embryos. Two specific bands in the *SAWPA* group were identified as HNRNPA1 and MED8, compared to the *SAWPA*‐rev control (**Figure** **5**A). To verify this result, western blotting analysis was carried out (Figure 5B). The results of IF and qPCR indicated that HNRNPA1 and MED8 exhibited higher expression levels at the 8‐cell stage (Figure 5C–E), consistent with the high expression level of *SAWPA* at the 8‐cell stage (Figure 2D). Furthermore, to confirm the *SAWPA* protein complex, a co‐immunoprecipitation (co‐IP) assay was conducted using 8‐cell embryos overexpressing HA‐tagged MS2 protein and MS2‐tagged *SAWPA*. The co‐IP results using an anti‐HA antibody (with IgG as a control) demonstrated that *SAWPA* indeed interacts with HNRNPA1 and MED8 to form an RNA‐protein complex (Figure 5F). MED8 is an essential component of the mediator complex, which acts as a coactivator to regulate the transcription of almost all RNA polymerase II‐dependent genes.^[^ ^26^ ^]^ As part of this complex, MED8 interacts with RNA polymerase II and gene‐specific transcription factors to facilitate transcriptional activation.^[^ ^26^, ^27^ ^]^ HNRNPA1 is a typical RNA‐binding protein that plays a significant role in transcription and alternative splicing. HNRNPA1 can interact with various non‐coding RNAs to exert its functions, participating in neurodegenerative diseases and cancer‐related conditions.^[^ ^28^ ^]^ These findings also suggest that *SAWPA* interacts with HNRNPA1 and MED8 to execute its function.

### 2.7 SINE Is the Key Sequence for the Functioning of *SAWPA*

**Figure 6:**
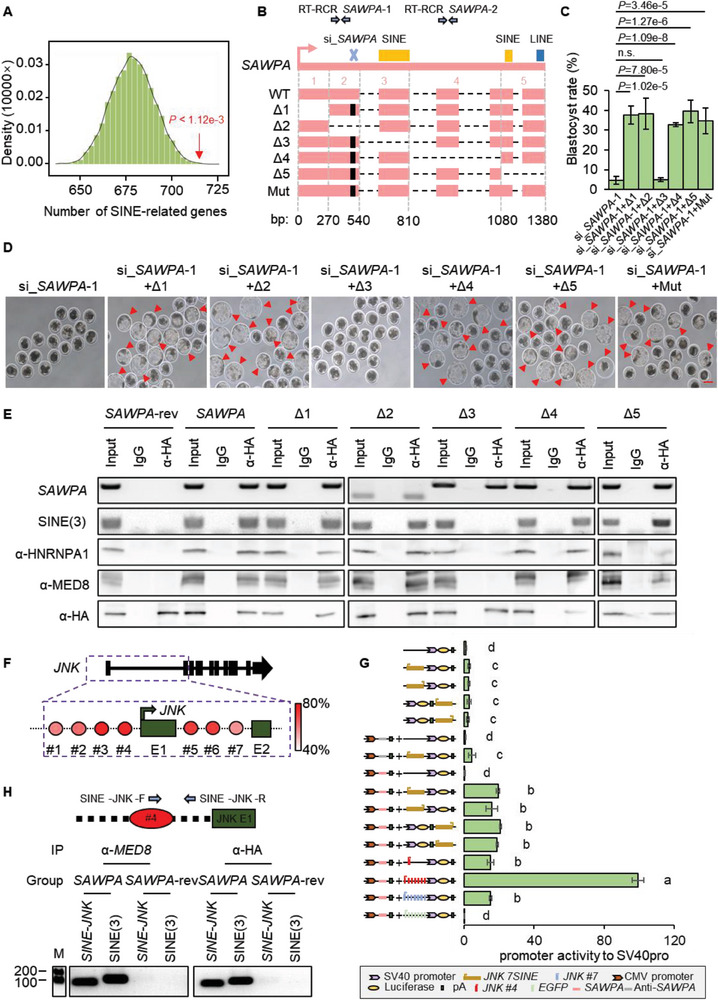
*SAWPA* regulates *JNK* transcription by binding to MED8 and HNRNPA1 through SINE sequences. A) The SINE‐associated genes of DEGs (red arrow) and the number of SINE‐associated random genes (black) (*P* < 1.12e‐3) measured using the Wilcoxon rank single test. SINE‐associated genes are gene loci that contain SINE elements. The interference with *SAWPA* was more likely to affect SINE‐associated genes. B) Experimental scheme to analyze the truncated sequence of *SAWPA*. Mut, *SAWPA* with mutation of si_*SAWPA*‐1 site. WT, wild type; RT‐PCR *SAWPA*; The RT‐PCR primer of *SAWPA* C) Blastocyst rate of the overexpressed six RNA fragments upon *SAWPA* depletion. si_*SAWPA*‐1 *and* si_*SAWPA*‐1 *+* Δ3 OE significantly decreased blastocyst rate. Three experimental replicates were used. Two‐tailed Student's *t*‐test was used for statistical analysis, and the error bars represent S.E.M. D) Photographs of the overexpressed six RNA fragments upon *SAWPA* depletion. The photographs were taken at pPA 168 h at the blastocyst stage. Embryos injected with si_*SAWPA*‐1 *+* Δ1 OE, si_*SAWPA*‐1 *+* Δ2 OE, OE, si_*SAWPA*‐1 *+* Δ4 OE, si_*SAWPA*‐1 *+* Δ5 OE, and si_*SAWPA*‐1 *+* Mut OE can develop to the blastocyst stage, while si_*SAWPA*‐1 *and* si_*SAWPA*‐1 *+* Δ3 OE embryos are arrested at the 8‐cell stage. Scale bar, 100 µm. At least three experimental replicates were used for each RNAi injection E) Co‐IP results in porcine PK15 using anti‐HA (for HA‐labeled MS2 coat protein). *SAWPA* forms an RNA–protein complex with HNRNPA1 and MED8. *SAWPA*‐rev, antisense sequence of *SAWPA* overexpression; *SAWPA*, *SAWPA* overexpression; The primer of RT‐PCR *SAWPA*‐1 were used to test *SAWPA* except Δ2 (RT‐PCR *SAWPA*‐2 were used); SINE(3), The SINE element of *SAWPA*; α‐, anti. For each co‐IP assay, ≈1 × 10^6^ porcine PK15s were used, and three experimental replicates were performed. F) Correlation between the *SAWPA* sequence and the *JNK* sequence. Complementary information of the sequence of exon 2 of *SAWPA* and the SINE sequence of the *JNK* promoter region. The SINE element located upstream and downstream of the TSS site in the *JNK* promoter region is named #1‐7. Different colors represent the degree of base matching; E1, exon1. G) *SAWPA* dual‐luciferase reporter system shows that *SAWPA* increased the enhancer activity of SINE in PK15 cells in a special sequence. The enhancer activity of SINE is increased by overexpression of *SAWPA*. The *y*‐axis shows the construction of luciferase reporter plasmids and overexpressed genes. Three experimental replicates were used. Two‐tailed Student's *t*‐test was used for the statistical analysis. Different letters indicate a significant difference (*p* < 0.01). H) Co‐IP followed by PCR assays results in porcine PK15 using anti‐MED8 and anti‐HA (for HA‐labeled MS2 coat protein). *SAWPA*, *SAWPA* overexpression; *SAWPA*‐rev, antisense sequence of *SAWPA* overexpression; SINE‐JNK, The PCR products were composed of SINE sequence (#4) and JNK; SINE(3), DNA sequence of 3 (SINE element of *SAWPA*); JNK E1, exon1 of JNK. Three experimental replicates were used.

SINE acts as a regulator, regulating gene expression through epigenetic mechanisms or directly as a binding site for distal enhancers, promoters, and transcription factors.^[^ ^29^ ^]^ In order to investigate if *SAWPA* regulates downstream genes through SINE elements, we initially defined SINE‐associated genes as those harboring a SINE sequence within 2 kb upstream and 1 kb downstream of the transcription start site (TSS). We then observed that out of 875 DEGs, 714 were SINE‐associated genes. To determine the statistical significance of this number, we performed a Wilcoxon rank‐sum test by comparing the observed number of SINE‐associated genes in DEGs to 875 genes randomly selected from the entire genome in 10 000 trials. The rank of DEGs was significantly higher than the distribution of 10 000 random controls (5% = 697, *p* < 1.12e‐3), indicating that interference with *SAWPA* was more likely to affect SINE‐associated genes (**Figure** **6**A).

To investigate the key sequences involved in the regulatory mechanism of *SAWPA*, we generated five *SAWPA* truncated mutations (from Δ1 to Δ5). To avoid the influence of the si_*SAWPA*‐1 site on the *SAWPA* sequence, we mutated the si_*SAWPA*‐1 sites in each truncated mutation, and mutated si_*SAWPA*‐1 sites in *SAWPA* as the Mut control group (Figure 6B). Next, we divided the embryos into six groups and overexpressed six RNA fragments upon *SAWPA* depletion (si_*SAWPA*‐1 + Δ1 OE, si_*SAWPA*‐1 + Δ2 OE, si_*SAWPA*‐1 + Δ3 OE, si_*SAWPA*‐1 + Δ4 OE, si_*SAWPA*‐1 + Δ5 OE, and si_*SAWPA*‐1 + Mut OE). The development data revealed that injection of Δ1, Δ2, Δ4, Δ5, and Mut, but not Δ3, can rescue the development arrest upon *SAWPA* depletion, (Figure 6C,D and **Table** **2**). This suggests that 3 (540–810 bp of *SAWPA*) is the critical domain for the regulatory function of *SAWPA*. As 3 contains the SINE sequence Pre0‐SS (the exon2 of *SAWPA*), we hypothesized that *SAWPA* exerts its regulatory function through the SINE element. To validate this hypothesis, we conducted an experiment on PK15 cells. We overexpressed the five truncated mations tagged with MS2 and HA‐tagged MS2 protein and performed co‐IP assays using HA antibodies. The results showed that Δ1, Δ2, Δ4, and Δ5 could bind to HNRNPA1 and MED8 through the 3 sequence, whereas Δ3, which lacks the SINE element, could not bind to HNRNPA1 and MED8 (Figure 6E). Next, we overexpressed *SAWPA*‐MS2 and HA‐MS2P at the 1‐cell stage of embryo and PK15, then performed co‐IP followed by RT‐PCR (test *SAWPA*) and PCR (test SINE (3): DNA sequence of 3) assays at the 8‐cell stage (Figure 5F) and PK15 (Figure 6E), we successfully detected the presence of *SAWPA* and SINE (3) element. This indicates that *SAWPA* exerts its regulatory function by binding to HNRNPA1 and MED8 through the SINE element. Therefore, our results demonstrate that *SAWPA* can interact with MED8 and HNRNPA1 to form RNA‐protein complexes through the SINE sequence, playing a crucial role in the early embryonic development and ZGA process in pigs.

**Table 2:** Embryonic development about *SAWPA* depletion with different lengths of *SAWPA* overexpression.

	Experiments [n]	Embryos [n]	BL [%]	2C arrest [%]	8C arrest [%]	4–8C arrest [%]
si_*SAWPA*‐1‐1	7	209	4.5 ± 1.9	8.7 ± 4.6	62.0 ± 2.0	80.5 ± 6.0
si_*SAWPA*‐1 + Δ1	5	163	37.7 ± 4.5	7.7 ± 2.0	22.0 ± 4.7	33.8 ± 6.5
si_*SAWPA*‐1 + Δ2	5	182	38.3 ± 8.0	4.1 ± 3.0	22.9 ± 3.2	33.3 ± 6.5
si_*SAWPA*‐1 + Δ3	6	164	5.0 ± 1.1	12.4 ± 2.1	48.9 ± 1.6	60.5 ± 3.3
si_*SAWPA*‐1 + Δ4	5	169	32.7 ± 1.0	9.1 ± 4.0	23.0 ± 5.0	40.5 ± 6.5
si_*SAWPA*‐1 + Δ5	6	250	39.4 ± 5.6	3.8 ± 2.3	21.5 ± 2.5	36.8 ± 4.3
si_*SAWPA*‐1 + Mut	4	119	34.7 ± 6.5	10.9 ± 2.9	26.0 ± 3.2	39.4 ± 1.0

8C, 8‐cell stage; 4–8C, 4‐ to 8‐cell stage; BL, blastocyst stage; si_, siRNA. Differences of data [mean ± standard error of the mean (s.e.m.)] were analyzed by using a two‐tailed Student's *t*‐test. Specific *p*‐values can be found in Table S6 (Supporting Information).

### 2.8 *SAWPA* Regulates JNK Transcription Through SINE Sequences

To investigate the mechanism by which *SAWPA* regulates *JNK*, we analyzed the sequences of *SAWPA* and *JNK*. The SINE‐associated element sequences of *SAWPA* highly coincided with the SINE sequences of *JNK*, particularly those located near the transcription start site (TSS), which had an overlapping region of ≈200 bp (Figure 6F; Figure S6, Supporting Information). This suggested that *SAWPA* regulates the transcription of *JNK*. For ease of reference, we named the SINE sequences upstream and downstream of the *JNK* TSS site as SINE1‐7, in their sequential order. SINE4 (#4) showed a high degree of overlap with exon 2 of *SAWPA* (Figure 6F).

TEs play an important role in transcription by regulating the transcription of related genes in both *cis*‐ and *trans‐*acting forms.^[^ ^9^, ^12^, ^16^ ^]^ Long non‐coding RNA can act as a scaffold and play a critical role in transcription. TEs‐associated lincRNAs act as enhancers in transcription.^[^ ^30^ ^]^ Therefore, we hypothesis that *SAWPA* may bind to related SINE elements and mediate its *cis*‐regulatory activity in *trans*. We confirmed this hypothesis through a double luciferase experiment to detect whether *SAWPA* increased the SV40 promoter activity in PK15 cells by a specific JNK SINE sequence. First, we found that 7SINE (SINE1∼7) of JNK can increase fluorescence intensity, suggesting that 7SINE of JNK has an enhancer effect, but the specific mechanism remains unclear (Figure 6G). Next, the result shows that overexpression of *SAWPA* increased the SV40 promoter activity by *JNK* 7SINE, regardless of whether the sense and antisense 7SINE of JNK were inserted upstream or downstream of the pGL3‐luciferase vectors (Figure 6G). Overexpression of *SAWPA* and *JNK* SINE4 resulted in fluorescence intensity of the pGL3‐luciferase vector that was similar to that of *JNK* 7SINE. This indicated that SINE4 was the key sequence of *SAWPA* binds to JNK. In addition, Overexpression of 7x *JNK* SINE4 and *SAWPA* led to further increases in the fluorescence intensity of the pGL3‐luciferase vector, while 7x *JNK* SINE7 and 7x EGFP (same length as 7x *JNK* SINE4) and *SAWPA* did not achieve the same fluorescence intensity (Figure 6G). Therefore, *SAWPA* may act as a transcription factor by binding to SINE and enhancing *cis* adjustment activity of SINE in the *JNK* promoter region by binding to SINE4.

**Figure 7:**
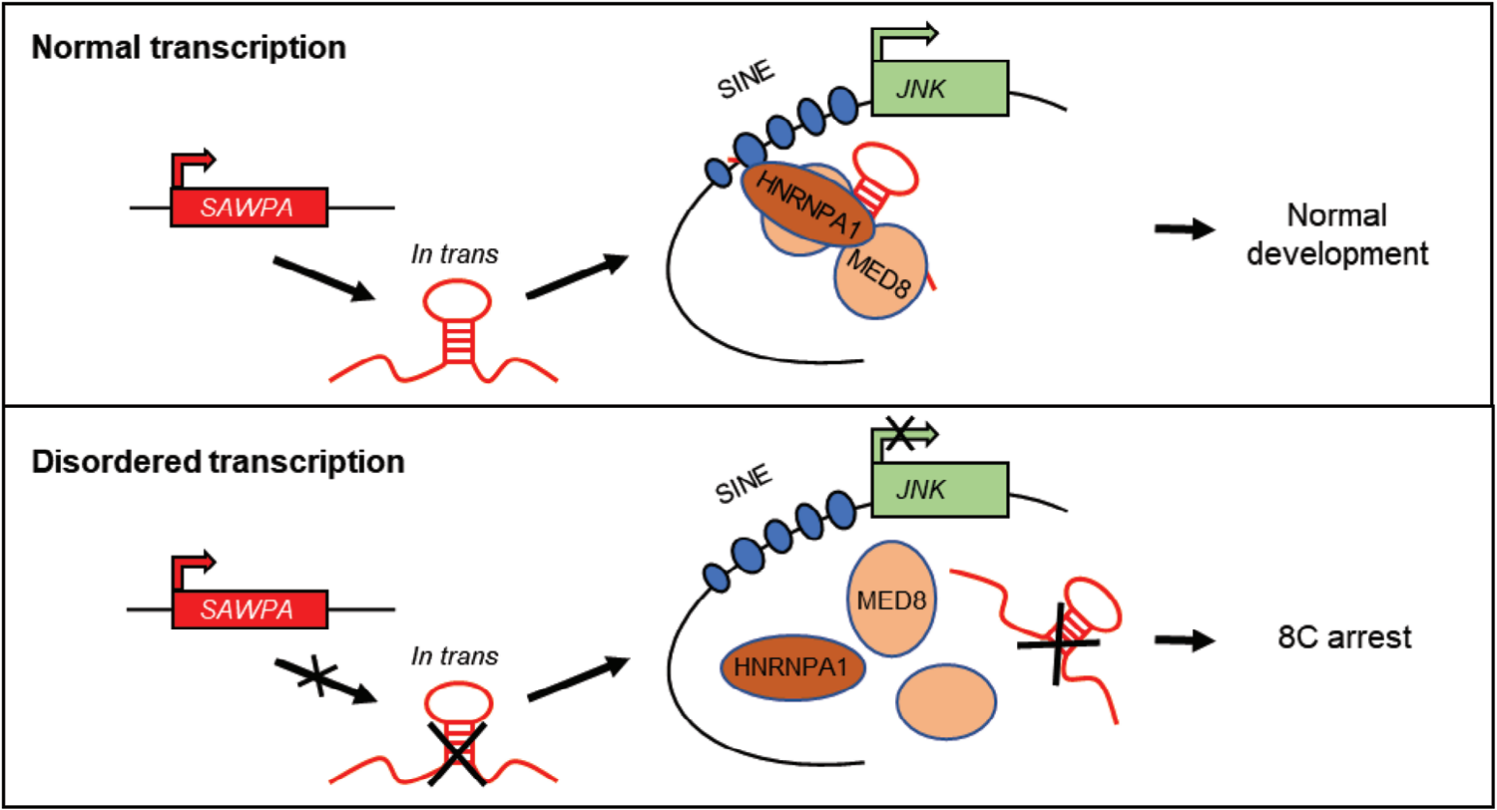
Model showing that *SAWPA* regulates *JNK* by binding to MED8 and HNRNPA1 through the SINE sequence.

To further confirm that the RNA‐protein complex is associated with JNK by SINE sequence, we performed co‐IP followed by PCR assay with HA‐tagged MS2‐labeled *SAWPA* overexpression in PK15 cells. The co‐IP followed by PCR assay (The SINE – *JNK* primers consist of a SINE sequence (#4) and a *JNK* promoter region) results using anti‐MED8 and anti‐HA antibodies showed that *SAWPA* can bind to JNK promoter sequences (Figure 6H). In summary, *SAWPA* can act as a transcription factor to *tran*s‐activate target genes through its reverse action on *cis*‐regulatory elements. *SAWPA* activates target gene expression by binding to SINE elements in the *JNK* promoter region and transactivating *JNK* transcription through interaction with HNRNPA1 and MED8 in *trans* (**Figure** **7**).

## 3 Discussion

In this study, we identified a SINE‐associated lncRNA in porcine preimplantation embryos and named it *SAWPA*. *SAWPA* is 8‐cell‐specific and localizes to the nucleus. Interference of *SAWPA* leads to embryonic developmental arrest at the 8‐cell stage; in addition, it restricts the opening of chromatin and the activation of ZGA. Mechanistically, *SAWPA* forms a complex with HNRNPA1 and MED8 and binds SINE elements in the promoter of *JNK* to activate its transcription, regulating the development through JNK‐MAPK signaling pathway. For the first time, we found that TEs‐associated lncRNA functions in pig early embryonic development, providing a new perspective for mechanism research on porcine‐specific early embryonic development.

The activity of TEs gradually increases during early mammalian embryonic development; it plays an important regulatory role in early embryonic development.^[^ ^31^ ^]^ MERVL is highly activated in mouse 2‐cell embryos, resulting in large amounts of ERV‐associated chimeric transcripts.^[^ ^31^, ^32^ ^]^ Based on those 2‐cell‐specific chimeric transcripts, 2C‐like embryonic stem cells were identified,^[^ ^33^ ^]^ indicating that the activation of ERVs is correlated with the acquisition of pluripotency both in early embryos and in stem cells. In humans, the activation of ERVK is associated with blastocyst pluripotency; HERVK is a marker for naïve‐like human ES cells.^[^ ^33^, ^34^ ^]^ Despite the appearance of a large number of TE element transcripts during early embryonic development, their regulatory mechanisms remain unclear. However, SINE can affect gene transcription.^[^ ^18^, ^35^ ^]^ During transcription, SINEs are spread throughout the genome, and their copies can be inserted into gene regions, where they can bind to nuclear proteins or become part of the gene regulatory system through modification, regulating gene transcription. SINE sequences provide multiple protein‐binding sites that mediate the transcription of RNA polymerase II. SINE B1 and SINE B2 were found in mice under heat shock reaction conditions. After heat shock, polymerase III specifically enhances the transcription of SINE B2 and Alu, which then binds to polymerase II, and inhibits the transcription of specific genes, such as actin and histone H1 genes, in a trans‐manner.^[^ ^36^ ^]^ Besides, the degradation of SINE B2 is also involved in upregulating stress genes in heat shock stress response, where EZH2 is recruited to trigger the cleavage of SINE B2.^[^ ^37^ ^]^ Here, we determined that the SINE‐associated *SAWPA* can bind to MED8, a component of Mediator, and then activate the transcription of target genes including *JNK*, These findings have expanded our understanding of the mechanisms regulating transcription of SINE‐associated sequences.

SINE‐associated lncRNA could be involved in transcriptional regulation.^[^ ^38^ ^]^ TEs can regulate transcription by binding lncRNAs; the ERV‐associated lncRNA, *LincGET* regulates transcription by mediating the *cis*‐regulatory activity of GLKLTRs and ZGA‐related genes.^[^ ^9^, ^30^ ^]^ The expression of LINE1 elements increases chromatin accessibility.^[^ ^11^ ^]^ *SAWPA* can mediate the regulatory activity of SINE elements to regulate the expression of target genes by binding HNRNPA1 and MED8, providing a *trans*‐regulatory model for SINE elements. SINE elements are widely distributed throughout the genome; therefore, SINE‐associated lncRNA is likely that their target genes are also widely distributed. Therefore, cut&tag could be used to detect whether *SAWPA* target genes play a key role in porcine‐specific early embryonic development on a genome‐wide scale.

Heterogeneous nuclear RNA‐protein complex (hnRNP) is well‐known to mediate the function of lncRNAs. For example, in mice, hnRNPU can interact with *LincGET* to produce regulatory effects in early embryonic development^[^ ^9^ ^]^; hnRNPK can bind to *Neat1* to mediate paraspeckles formation^[^ ^39^ ^]^; hnRNPK interacts with *Xist* to regulate chromatin diffusion, gene silencing, and expression^[^ ^39^, ^40^ ^]^; hnRNPK interacts with lncRNAs containing SINE derived nuclear RNA localization (SIRLOIN) and mediates nuclear enrichment nuclear cell function.^[^ ^41^ ^]^ In this study, we found that HNRNPA1 can bind to SINE‐associated lncRNA *SAWPA* to regulate the transcription of JNK, reflecting a conserved mechanism of SINE elements in transcription regulation between mice and pigs.

John et al. used chromatin immunoprecipitation‐sequencing (ChIP‐seq) to show that lncRNAs are generally located at the junction between active and inactive chromatin during the early stages of embryonic development.^[^ ^42^ ^]^ Several lncRNAs can recruit epigenetic factors upon binding to target genes to regulate chromatin structure. The human lncRNA, HOTAIR interacts with PRC2 and LSD1‐CoREST‐REST complexes, inhibiting the expression of HoxD gene clusters by erasing H3K4me2/3 and establishing H3K27me3.^[^ ^42^, ^43^ ^]^ Similarly, human HOTTIP recruits MLL through WDR5 into the 5′ region of the HoxA gene cluster, catalyzing the establishment of H3K4me3 and *cis*‐activating the expression of genes such as Hoxa11 and Hoxa13. *LincGET* promotes the target gene methylation of histone H3 arginine 26 (H3R26me) by recruiting CARM1, which increases chromatin openness.^[^ ^44^ ^]^ The recruitment of epigenetic factors by lncRNAs provides a novel perspective. Therefore, we speculate that *SAWPA* may be involved in the recruitment of epigenetic factors that promote chromatin opening. Our future research will focus on detecting the epigenetic factors recruited by *SAWPA*, through IP mass spectrometry, to explore the specific mechanism of *SAWPA* regulation in early embryonic development.

This study identified that the JNK‐MAPK signaling pathway is crucial for early embryonic development in pigs. We observed that inhibiting *JNK* expression and phosphorylation hindered embryonic development, primarily at the 8‐cell stage. This finding aligns with the regulatory role of the MAPK signaling pathway in other species and cell types. In mice, the ERK‐MAPK signaling pathways affect ZGA, with inhibitors arresting embryos at the G2 stage of the 2‐cell stage.^[^ ^45^ ^]^ Inhibition of the p38‐MAPK pathway led to developmental arrest at the 8–16‐cell stage and inhibited blastocyst expansion and incubation.^[^ ^46^ ^]^ In pig embryo research, the MAPK signaling pathway is vital in the formation of the inner cell mass and the induction of ectodermal stem cells (EpiSCs).^[^ ^47^ ^]^ Therefore, the MAPK signaling pathway plays a crucial regulatory role in early pig embryos, reflecting the regulatory mechanisms involved in early pig embryo development. Although the specific mechanisms underlying the effects of the JNK signaling pathway are not yet clear, we will also focus our attention on the target genes affected by JNK in the future, in order to further investigate the root causes of blockages that occur.

In summary, our study revealed the crucial involvement of SINE‐associated lncRNAs during the ZGA of embryonic development, highlighting the indispensability of SINE, an endogenous retrovirus, in this process. However, the precise regulatory mechanisms behind this phenomenon are yet to be fully understood. Given the significant presence of SINE in animal genomes, investigating the connection between SINE‐associated lncRNAs and pluripotency or reprogramming in the future would be a valuable endeavor.

## 4 Experimental Section

### Primer and Probe Design

The primers were designed using PrimerPremier5 (Table S1, Supporting Information), and primers and FISH probes were synthesized by Beijing Genomics Institute (Beijing, China).

### Antibodies

The following antibodies were used for BrdU, western blot, and co‐IP assay: anti‐BrdU (#ab1893; Abcam), anti‐β‐actin (#sc47778; Santacruz), anti‐JNK1 + JNK2 + JNK3 (phospho‐T183 + T183 + T221) (#ab124956; Abcam), anti‐JNK1 + JNK2 + JNK3 (#ab179461; Abcam), anti‐MED8 (#PA5‐118854; Thermo), anti‐HBRNPA1 (#11176‐1‐AP; proteintech) and anti‐HA (#ab1424; Abcam).

### Embryo culture and collection

Pig ovaries were collected from the slaughterhouse and transported in 37 °C saline solution. Upon arrival, they were thoroughly rinsed with a physiological saline solution containing penicillin (S9137; Sigma) and streptomycin (P3032; Sigma) at a temperature of 37 °C. To obtain the cumulus‐oocyte complex (COCs) from follicles measuring 3–6 mm in diameter, a 10 mL syringe fitted with an 18‐gauge needle was used for aspiration. The COCs were then washed three times with a HEPES (H3784‐1KG; Sigma) buffer solution. High‐quality COCs were selected for in vitro mature. Fifty COCs were transferred to 500 µL of TCM‐199 culture medium (3100‐027; Invitrogen) supplemented with 0.14% PVA (P8136; Sigma), 10 ng mL−1 epidermal growth factor (E4127; Sigma), 0.57 mm cysteine (C7602; Sigma), 0.5 IU mL−1 pregnant mare serum gonadotropin (PMSG, hor272; PROSPEC), and 0.5 IU mL−1 human chorionic gonadotropin (hCG, hor250; PROSPEC). After being collected, the COCs were cultured at 39 °C until they matured, which took ≈42–44 h. Once mature, they were transferred to an Eppendorf tube (MCT‐150‐C; AXYGEN) containing 0.1% hyaluronidase, and the surrounding cumulus cells were manually removed using a pipette. COCs with visible polar bodies were then selected for parthenogenetic activation (PA). The parthenogenetically activated embryos were cultured in PZM3 medium, which was 500 µL PZM‐3 containing 0.15% BSA (A8022; Sigma), 200 µL BME (B6766; Merck) Amino Acids Solution, and 100 µL MEM (M7145; Merck) with no essential amino acid solution.

Post parthenogenetic activation (pPA), embryos were collected at different time points: 1‐cell stage (pPA 12 h), 2‐cell stage (pPA 24 h), early 4‐cell stage (pPA 40 h), middle 4‐cell stage (pPA 48 h), late 4‐cell stage (pPA 56 h), 8‐cell stage (pPA 72 h), morula stage (pPA 108 h), early blastocyst stage (pPA 120 h), middle blastocyst stage (pPA 144 h), and late blastocyst stage (pPA 168 h).

### RNA‐Seq and Analysis

Embryos at the 8‐cell stage were collected from the si_*SAWPA*‐1 and si_NC groups using TRIZOL. Each experiment involved 400 embryos and was repeated twice. The collected embryos were washed with PBSA at least three times and PBSA was removed as thoroughly as possible. The Beijing Genomics Institute performed the RNA extraction, quality control, library construction, and sequencing of the embryo samples using the DNBSEQ Low Input Smart‐Seq Eukaryotic mRNA library, following the manufacturer's instructions.

### Analysis of TEs‐Associated LncRNA

In data quality control, first, RNA‐seq data were downloaded from public databases with the following data identifiers: GSE163620, CRA004237, GSE138760, and GSE36552. Trim_galore(v0.6.7) was used to remove adapters and low‐quality sequences from the downloaded raw data, using default parameters. The resulting clean data were used for subsequent analysis.

### Prediction of LncRNA Transcripts

First, STAR (v2.7.10b) was used to align the clean data to the genome (using parameters: –outSAMstrandField intronMotif –outFilterIntronMotifs RemoveNoncanonical –outFilterMismatchNmax 2 –outFilterMultimapNmax 20 –outFilterMatchNmin 16 –alignEndsType EndToEnd –runThreadN 20 –outSAMtype BAM SortedByCoordinate –outBAMsortingThreadN 10). Then transcript assembly was performed using stringtie(v2.1.7) with default parameters. The assembled transcripts were compared to the genome annotation file using gffcompare (v0.12.6) (using default parameters). Transcripts with tags “u,” “x,” “i,” “j,” or “o,” a sequence length greater than or equal to 200, and containing at least 2 exons were selected as candidate transcripts for new lncRNAs. Subsequently, CPC2 (v0.1) (with default parameters), CNCI(v2) (with parameter ‐m ve), and PLEK (v1.2) (with default parameters) were used to predict the coding potential of the new transcripts. Transcripts, for which all three software tools predicted “nocoding”, were extracted. Finally, cufflinks (v2.2.1) were used for expression quantification of the predicted new lncRNA transcripts (using the parameter –library‐type fr‐unstranded). The quantified results were used for further analysis.

### Analysis of LncRNA‐Repeat Associations

Repeat element annotation files were downloaded from UCSC (pig: https://hgdownload.soe.ucsc.edu/goldenPath/susScr3/database/rmsk.txt.gz. mouse: https://hgdownload.soe.ucsc.edu/goldenPath/mm39/database/rmsk.txt.gz. human: https://hgdownload.soe.ucsc.edu/goldenPath/hg38/database/rmsk.txt.gz) and lncRNAs were classified into four types based on the types of repetitive elements found within their exons: SINE‐, LINE‐, ERVL‐, and other repeat‐ associated elements. Then the expression patterns of these different types of lncRNAs during embryonic development were analyzed.

### Analysis of ZGA‐Associated LncRNAs

The functions of significantly upregulated ZGA‐associated lncRNA and mRNA transcripts during the ZGA period were explored. Differential gene analysis was performed using DESeq2 (R3.5) for the 8C/MII stage, with a selection criterion of padjust<0.01 and |log2FoldChange|>1 for lncRNAs and mRNAs as ZGA‐associated candidates. These candidates were used for subsequent ZGA‐lncRNA analysis, including the counting of different TE types.

### Quantitative Real‐Time PCR

Real‐time quantitative PCR was performed to detect the expression of genes using TB Green Premix Ex Taq (RR420A; Takara). Total RNA was extracted from 100 embryos using TRIZOL reagent (15 596 018; Ambion) and quality check analysis was performed. Reverse transcription was performed using RevertAid (M1631; Thermo) under the following conditions: 60 min at 42 °C, followed by 5 s at 85 °C. The cDNA was stored at −20 °C until use. For qPCR, the following conditions were used: 30 s at 95 °C, 40 cycles of 5 s at 95 °C, and 34 s at 60 °C, followed by a dissociation stage comprising 15 s at 95 °C and 1 min at 60 °C. The cycle threshold (Ct) value for each sample was obtained from three experimental replicates. The target sequence was normalized to the reference sequence using the 2^−ΔΔCt method.

### RNA‐FISH

The RNA fluorescence in situ hybridization (RNA‐FISH) was carried out following a previously established protocol.^[^ ^4^ ^]^ The probes were labeled through in vitro transcription using the MEGAshortscript Kit (AM1354; Ambion), and 75% of the uracil was labeled with Alexa Fluor 488 (C11403; Invitrogen) in a 4:1 ratio of Alexa Fluor 488‐5‐UTP to UTP. The vitelline membrane was removed with acidic treatment (10 µL HCl, 1 mL MAN) and the pig embryos were incubated in PBS containing 6 mg mL^−1^ BSA for 3 min. Subsequently, the embryos were transferred onto Superfrost/Plus microscope slides (12‐550‐15; Fisher) and dried immediately. The embryos were fixed and permeabilized with 4% paraformaldehyde (PFA). The D‐T‐G lncRNA in situ hybridization kit (D‐074; FOCO) was employed to detect the localization of *SAWPA* embryos, and imaging was performed using a confocal microscope (TCS SP8; Leica, Wetzlar, Germany).

### Microinjection

For lncRNA and mRNA downregulation/or overexpression, the RNA (si_RNA, 20 µm; mRNA, 150 ng µL^−1^) was injected into the cytoplasm of mature oocytes using a FemtoJet microinjector (Eppendorf; Hamburg, Germany), the same amount was injected into each embryo, and the injection conditions were 150 hPa injection pressure, 50 hPa compensation pressure, and 0.7 s injection time. The injection amount per embryo was ≈10 pL. The RNA was delivered into the porcine mature oocyte at 6 h after PA. The operation was performed on a heated stage of an inverted microscope (Nikon Corporation; Tokyo, Japan), and microinjection was carried out in MAN buffer medium.

### BrdU Staining

The embryos injected with si_RNA or si_NC were cultured in PZM‐3 after PA 6 h. After PA 66 h, BrdU was added at a final concentration of 20 µg mL^−1^ and aphidicolin at 0.5 µg mL^−1^. The embryos were cultured in PZM‐3 for 24 h, fixed for 90 h, and subjected to IF. Zona pellucida was removed at 90 h after PA, and the embryos were transferred into the acid operating solution (10 µL HCL in 1 mL MAN). The 8‐cell stage embryos were immediately cleaned in MAN thrice. The embryo was placed in a 4% PFA fixation solution and fixed at room temperature for 30 min. After three washes for 5 min each in PBSA (0.2 g BSA in 100 mL PBS), the embryos were incubated at room temperature for 30 min in 1.5 m HCl (concentrated hydrochloric acid:H2O = 1:7). The embryos were washed three times with PBSA, then they were subjected to permeabilization in a normal permeation solution, which involved dissolving 0.75 mL of TritonX‐100 (T9284‐100ML; Sigma) in 50 mL of PBS (P2272; Sigma). The embryos were transferred to 500 µL membrane permeable solution and permeated overnight at 4 °C for 8−12 h (0.75 mL TritonX‐100 in 50 mL PBS). The embryos were washed with PBSA twice (5 min/time). After incubating at room temperature for 1 h, the primary antibody was incubated overnight at 4 °C with a blocking solution (0.1 g BSA in 10 mL PBS) diluted. Following three washes in PBSA, the embryos were incubated with the secondary antibody diluted with blocking solution and incubated at room temperature and away from light for 2 h. Once the embryos were washed three times with the cleaning solution, they were stained with Hoechst33342 (10 ng µL^−1^ in 1× PBS, H3570, Invitrogen) for 8 min. Then, after washing three times with PBSA, the embryos were mounted on a glass slide, sealed, and visualized.

### EU Staining

At 56 h after PA, the pig embryos injected in si_NC or si_*SAWPA*‐1 groups were supplemented with EU to achieve a final concentration of 10 mm. The embryos were then cultured for an additional 16 h. The cumulus cells were removed using acidic Tyrode's solution, and the resulting 8‐cell stage embryos were washed twice. Next, the embryos were fixed with 4% PFA, washed three times with PBSA, and permeabilized with a permeabilization buffer. Subsequently, experiments were carried out using the click‐iT RNA Alexa Fluor 488 Imaging Kit (C10329; Invitrogen) to detect the levels of newly synthesized EU.

### In Vivo DNase I‐TUNEL Assay

The DNase I‐TUNEL assay in vivo was conducted as per a previously published article.^[^ ^4^ ^]^ si_*SAWPA*‐1 and si_NC were injected during the 1‐cell stage, and samples were collected at the 8‐cell stage of embryonic development. The embryos were washed twice with PBSA and then subjected to in vivo permeabilization on ice for 5 min using extraction buffer (50 mm NaCl, 3 mm MgCl2, 0.5% Triton X‐100, and 300 mm sucrose, 25 mm HEPES, pH 7.4). Afterward, the embryos were washed twice with extraction buffer without Triton X‐100 and incubated with 1 U mL^−1^ of DNase I (AM2222; Ambion) in the same buffer at 37 °C for 5 min. Following fixation, the TUNEL BrightRed Cell Apoptosis Detection Kit (A113‐01; Vazyme) was utilized to detect TUNEL cell apoptosis. TUNEL staining solution containing FITC‐conjugated staining solution and terminal deoxynucleotidyl transferase was prepared in accordance with the instructions. The embryos were washed three times with PBSA and treated with 1% Triton X‐100 in PBSA for 1 h. Subsequently, the embryos were incubated with a TUNEL staining solution at 38 °C for 1.5 h. After washing thrice with PBS, the samples were transferred to PBSA containing 10 ng µL^−1^ Hoechst33342 for 10 min, mounted on slides with an anti‐fading solution, and examined using a fluorescence inverted microscope (ECLIPSE Ti‐S; Nikon, Japan). The experiment was repeated three times.

### Western Blot Analysis

Each group consisted of 200 embryos at a corresponding developmental stage. The embryos were lysed for 2 h in 60 µL of lysis buffer containing 20 mm HEPES, 1 mm EDTA, 20 mm glycerol phosphate, 150 mm NaCl, 2 mm EGTA, 10% glycerol supplemented, and 1% Triton X‐100 with 0.6 µL PMSF (100 mm, ST506; Beyotime). The lysates were then boiled at 100 °C for 5 min. The resulting proteins were separated using a 12% ExpressPlus PAGE gel (TM0645; GenScript), transferred to nitrocellulose membranes (3 010 040 001; Millipore), and detected by immunoblotting. The membranes were blocked with 5% BSA in TBST at room temperature for 2 h and then incubated overnight at 4 °C with primary antibodies against JNK, pJNK, or β‐actin. The antibody dilution ratio was 1:1000. After washing with TBST, they were incubated with HRP‐conjugated secondary antibodies. The antibody dilution ratio was 1:2000. SuperSignal West Pico PLUS (34 577; Thermo) was used for chemiluminescent detection in Western blot analysis according to the manufacturer's instructions, and images were captured using MiniChemi (MiniChemi580; SAGECREATION, Beijing, China).

### Double Luciferase Assay

Porcine kidney epithelial cells, PK15, were cultured; the interference fragment and control group were transfected into PK15 cells and cultured for 24 h. The plasmid TK was co‐transfected with *SAWPA* promoter‐pGL3‐luciferase, CMV‐, *JNK* SINE‐associated pGL3‐luciferase, CMV‐, and cultured for 48 h, to ensure that the cell density did not exceed 95%. The culture medium was removed, and PBS was added and washed twice by gently shaking the plate. The fluorescence intensity was measured using the Dual‐Luciferase Reporter Assay System (E1910; Promega).

### RNA Pull‐Down assay

RNAs were in vitro‐transcribed with mMESSAGEmMACHINE T7 ULTRA Kit (AMB1345‐5; Ambion) and biotinylated with Pierce RNA 3″‐End Desthiobiotinylation Kit (20 163; Pierce) following the manufacturer's manual. A slot blot was performed to demonstrate that RNAs were efficiently biotinylated. Biotinylated RNAs (50 pmol) were heated at 85 °C for 2 min, immediately put on ice for at least 2 min, and an equal volume of RNA structure buffer (10 mm Tris pH 7.0, 0.1 m KCl, 10 mm MgCl_2_) was added. The samples were then shifted to RT for at least 20 min to allow proper secondary structure formation. Eight‐cell stage embryos (for pull‐down mass spectrum, 2000 embryos were used, and for pulldown Western blot, ≈500 embryos were used for each time) were digested with Pierce IP Lysis Buffer (87 787; Pierce) supplied with protease inhibitor cocktail (78 441; Pierce) according to the manufacturer's protocol. RNA pull‐down was performed by Pierce Magnetic RNA‐Protein Pull‐Down Kit (20 164; Pierce) according to the manufacturer's protocol. The retrieved protein was detected by mass spectrometry (24 600; Thermo) according to the manufacturer's protocol or Western blot test.

### Co‐Immunoprecipitation

First, HA‐MS2P and *SAWPA*‐24×MS2 vectors were transfected into PK15 cells by Lipofectamine LTX & PLUS Reagent (15 338 100; Thermo). In the co‐IP experiment, Pierce Crosslink Magnetic IP/co‐IP Kit (88 805; Thermo), which had HA antibodies crosslinked to the Pierce Protein A/G Magnetic Beads, was used. First, cells were collected and washed thoroughly with PBS before dissolving 10^6^ PK15 cells in 100 µL of IP lysis buffer. Next, 10 µL of the resulting lysate was used as input. The remaining lysate was then incubated overnight at 4 °C with Protein A/G beads that were conjugated with anti‐HA antibodies. The beads were washed twice using IP lysis buffer for 5 min each time, and subsequently, the beads were resuspended in 500 µL of ultrapure water while gently agitating. Hundred microliters of elution buffer was added to collect the supernatant containing the target antigen, which was then mixed with 15 µL of western blot sample buffer and incubated at boiling water for 5 min. Finally, MED8 and HNRNPA1 were detected through Western blot analysis.

### Statistical Analyses

The experimental data were subjected to statistical analysis using EXCEL and GraphPad Prism 5.0 software and presented as means ± S.E.M. Between‐group comparisons were conducted using *t*‐tests or Wilcoxon rank sum tests, and the significance level was indicated by *p*‐values.

## Conflict of Interest

The authors declare no conflict of interest.

## Author Contributions

T.H., J.W., Z.L., and J.‐X.J. conceived and designed the study. T.H. and D.L. performed porcine embryo collection. T.H. performed porcine embryo experiments with contributions from J.P., S.Y., D.L., S.G., Y.Z., and Z.C. T.H. performed molecule associated experiments with contributions from S.G. and Z.C. T.H. performed cell associated experiments with contributions from Y.Z. and R.W. T.H. and Y.Z. analyzed the data with contributions from J.W. J.‐X.J. and B.C.L. supervised the project. T.H. and J.W. wrote the manuscript.

## Supporting information

Supporting Information

## Data Availability

The data that support the findings of this study are available from the corresponding author upon reasonable request.

## References

[1] a) W. Wang , L. Min , X. Qiu , X. Wu , C. Liu , J. Ma , D. Zhang , L. Zhu , Front. Cell Dev. Biol. 2021, 9, 645647; 34178980 10.3389/fcell.2021.645647 PMC8222981

[2] a) P. A. Latos , F. M. Pauler , M. V. Koerner , H. B. Senergin , Q. J. Hudson , R. R. Stocsits , W. Allhoff , S. H. Stricker , R. M. Klement , K. E. Warczok , K. Aumayr , P. Pasierbek , D. P. Barlow , Science 2012, 338, 1469; 23239737 10.1126/science.1228110

[3] N. Hamazaki , M. Uesaka , K. Nakashima , K. Agata , T. Imamura , Development 2015, 142, 910. 25633350 10.1242/dev.116996 PMC4352986

[4] J. Wang , L. Wang , G. Feng , Y. Wang , Y. Li , X. Li , C. Liu , G. Jiao , C. Huang , J. Shi , T. Zhou , Q. Chen , Z. Liu , W. Li , Q. Zhou , Cell 2018, 175, 1887. 30550787 10.1016/j.cell.2018.11.039

[5] M. G. Kidwell , Genetica 2002, 115, 49. 12188048 10.1023/a:1016072014259

[6] G. Bourque , K. H. Burns , M. Gehring , V. Gorbunova , A. Seluanov , M. Hammell , M. Imbeault , Z. Izsvák , H. L. Levin , T. S. Macfarlan , D. L. Mager , C. Feschotte , Genome Biol. 2018, 19, 199. 30454069 10.1186/s13059-018-1577-z PMC6240941

[7] a) H. M. Rowe , D. Trono , Virology 2011, 411, 273; 21251689 10.1016/j.virol.2010.12.007

[8] D. Kelley , J. Rinn , Genome Biol. 2012, 13, R107. 23181609 10.1186/gb-2012-13-11-r107 PMC3580499

[9] J. Wang , X. Li , L. Wang , J. Li , Y. Zhao , G. Bou , Y. Li , G. Jiao , X. Shen , R. Wei , S. Liu , B. Xie , L. Lei , W. Li , Q. Zhou , Z. Liu , EMBO Rep. 2016, 17, 1452. 27496889 10.15252/embr.201642051 PMC5048373

[10] F. Chen , M. Zhang , X. Feng , X. Li , H. Sun , X. Lu , Stem Cells Int 2021, 1, 6657597. 10.1155/2021/6657597 PMC7884122 33628268

[11] J. W. Jachowicz , X. Bing , J. Pontabry , A. Boskovic , O. J. Rando , M.‐E. Torres‐Padilla , Nat. Genet. 2017, 49, 1502. 28846101 10.1038/ng.3945

[12] J. Gassler , W. Kobayashi , I. Gáspár , S. Ruangroengkulrith , A. Mohanan , L. Gómez Hernández , P. Kravchenko , M. Kümmecke , A. Lalic , N. Rifel , R. J. Ashburn , M. Zaczek , A. Vallot , L. Cuenca Rico , S. Ladstätter , K. Tachibana , Science 2022, 378, 1305. 36423263 10.1126/science.abn7478

[13] a) U. A. Ørom , R. Shiekhattar , Trends Genet. 2011, 27, 433; 21831473 10.1016/j.tig.2011.06.009 PMC4484734

[14] a) X. Wang , S. Arai , X. Song , D. Reichart , K. Du , G. Pascual , P. Tempst , M. G. Rosenfeld , C. K. Glass , R. Kurokawa , Nature 2008, 454, 126; 18509338 10.1038/nature06992 PMC2823488

[15] a) P. Lefevre , J. Witham , C. E. Lacroix , P. N. Cockerill , C. Bonifer , Mol. Cell 2008, 32, 129; 18851839 10.1016/j.molcel.2008.07.023 PMC2581490

[16] a) S. Garbo , M. Tripodi , C. Battistelli , Oncoscience 2022, 9, 49; 36110328 10.18632/oncoscience.563 PMC9469907

[17] a) F. Chen , X. Li , X. Feng , T. Gao , W. Zhang , Z. Cheng , X. Zhao , R. Chen , X. Lu , Stem Cells 2022, 40, 1094; 36087098 10.1093/stmcls/sxac067

[18] a) M. Cowley , R. J. Oakey , PLoS Genet. 2013, 9, e1003234; 23358118 10.1371/journal.pgen.1003234 PMC3554611

[19] E. G. Park , H. Ha , D. H. Lee , W. R. Kim , Y. J. Lee , W. H. Bae , H.‐S. Kim , Int. J. Mol. Sci. 2022, 23, 8950.36012216

[20] a) R. A. Schoenbeck , M. S. Peters , L. F. Rickords , T. T. Stumpf , R. S. Prather , Biol. Reprod. 1992, 47, 1118; 1493177 10.1095/biolreprod47.6.1118

[21] a) P. Braude , V. Bolton , S. Moore , Nature 1988, 332, 459; 3352746 10.1038/332459a0

[22] B. Zhang , H. Zheng , B. Huang , W. Li , Y. Xiang , X. Peng , J. Ming , X. Wu , Y. Zhang , Q. Xu , W. Liu , X. Kou , Y. Zhao , W. He , C. Li , B. Chen , Y. Li , Q. Wang , J. Ma , Q. Yin , K. Kee , A. Meng , S. Gao , F. Xu , J. Na , W. Xie , Nature 2016, 537, 553. 27626382 10.1038/nature19361

[23] a) W. Tadros , H. D. Lipshitz , Development 2009, 136, 3033; 19700615 10.1242/dev.033183

[24] a) W. Zhou , Y.‐J. Niu , Z.‐W. Nie , J.‐Y. Kim , Y.‐N. Xu , C.‐G. Yan , X.‐S. Cui , Biochim. Biophys. Acta, Mol. Cell Res. 2020, 1867, 118648; 31935425 10.1016/j.bbamcr.2020.118648

[25] a) F. Lu , Y. Liu , A. Inoue , T. Suzuki , K. Zhao , Y. Zhang , Cell 2016, 165, 1375; 27259149 10.1016/j.cell.2016.05.050 PMC6625655

[26] a) S. Malik , R. G. Roeder , Trends Biochem. Sci. 2000, 25, 277; 10838567 10.1016/s0968-0004(00)01596-6

[27] a) R. D. Kornberg , Trends Biochem. Sci. 2005, 30, 235; 15896740 10.1016/j.tibs.2005.03.011

[28] a) I. Beusch , P. Barraud , A. Moursy , A. Cléry , F. H.‐T. Allain , Elife 2017, 6, 25736; 10.7554/eLife.25736 PMC5503513 28650318

[29] D. A. Kramerov , N. S. Vassetzky , Wiley Interdiscip. Rev. RNA 2011, 2, 772. 21976282 10.1002/wrna.91

[30] a) J. Deforges , R. S. Reis , P. Jacquet , D. J. Vuarambon , Y. Poirier , BMC Genomics 2019, 20, 601; 31331261 10.1186/s12864-019-5946-0 PMC6647327

[31] a) A. E. Peaston , A. V. Evsikov , J. H. Graber , W. N. De Vries , A. E. Holbrook , D. Solter , B. B. Knowles , Dev. Cell 2004, 7, 597; 15469847 10.1016/j.devcel.2004.09.004

[32] A. V. Evsikov , W. N. De Vries , A. E. Peaston , E. E. Radford , K. S. Fancher , F. H. Chen , J. A. Blake , C. J. Bult , K. E. Latham , D. Solter , B. B. Knowles , Cytogenet. Genome Res. 2004, 105, 240. 15237213 10.1159/000078195

[33] T. S. Macfarlan , W. D. Gifford , S. Driscoll , K. Lettieri , H. M. Rowe , D. Bonanomi , A. Firth , O. Singer , D. Trono , S. L. Pfaff , Nature 2012, 487, 57. 22722858 10.1038/nature11244 PMC3395470

[34] a) E. J. Grow , R. A. Flynn , S. L. Chavez , N. L. Bayless , M. Wossidlo , D. J. Wesche , L. Martin , C. B. Ware , C. A. Blish , H. Y. Chang , R. A. Reijo Pera , J. Wysocka , Nature 2015, 522, 221; 25896322 10.1038/nature14308 PMC4503379

[35] P. Yakovchuk , J. A. Goodrich , J. F. Kugel , Proc. Natl. Acad. Sci. U. S. A. 2009, 106, 5569. 19307572 10.1073/pnas.0810738106 PMC2667051

[36] a) T. A. Allen , S. Von Kaenel , J. A. Goodrich , J. F. Kugel , Nat. Struct. Mol. Biol. 2004, 11, 816; 15300240 10.1038/nsmb813

[37] A. Zovoilis , C. Cifuentes‐Rojas , H.‐P. Chu , A. J. Hernandez , J. T. Lee , Cell 2016, 167, 1788. 27984727 10.1016/j.cell.2016.11.041 PMC5552366

[38] a) A. T. Brini , G. M. Lee , J. P. Kinet , J. Biol. Chem. 1993, 268, 1355; 8419337

[39] a) T. Naganuma , S. Nakagawa , A. Tanigawa , Y. F. Sasaki , N. Goshima , T. Hirose , EMBO J. 2012, 31, 4020; 22960638 10.1038/emboj.2012.251 PMC3474925

[40] a) N. Jansz , T. Nesterova , A. Keniry , M. Iminitoff , P. F. Hickey , G. Pintacuda , O. Masui , S. Kobelke , N. Geoghegan , K. A. Breslin , T. A. Willson , K. Rogers , G. F. Kay , A. H. Fox , H. Koseki , N. Brockdorff , J. M. Murphy , M. E. Blewitt , Cell Rep. 2018, 25, 1912; 30428357 10.1016/j.celrep.2018.10.044

[41] S. Zhu , Z. Wang , J. Xu , Trends Biochem. Sci. 2019, 44, 733. 31279651 10.1016/j.tibs.2019.06.001

[42] J. L. Rinn , M. Kertesz , J. K. Wang , S. L. Squazzo , X. Xu , S. A. Brugmann , L. H. Goodnough , J. A. Helms , P. J. Farnham , E. Segal , H. Y. Chang , Cell 2007, 129, 1311. 17604720 10.1016/j.cell.2007.05.022 PMC2084369

[43] M.‐C. Tsai , O. Manor , Y. Wan , N. Mosammaparast , J. K. Wang , F. Lan , Y. Shi , E. Segal , H. Y. Chang , Science 2010, 329, 689. 20616235 10.1126/science.1192002 PMC2967777

[44] K. C. Wang , Y. W. Yang , B. Liu , A. Sanyal , R. Corces‐Zimmerman , Y. Chen , B. R. Lajoie , A. Protacio , R. A. Flynn , R. A. Gupta , J. Wysocka , M. Lei , J. Dekker , J. A. Helms , H. Y. Chang , Nature 2011, 472, 120. 21423168 10.1038/nature09819 PMC3670758

[45] M. Maekawa , T. Yamamoto , M. Kohno , M. Takeichi , E. Nishida , Development 2007, 134, 2751. 17611221 10.1242/dev.003756

[46] a) D. R. Natale , A. J. M. Paliga , F. Beier , S. J. A. D'souza , A. J. Watson , Dev. Biol. 2004, 268, 76; 15031106 10.1016/j.ydbio.2003.12.011

[47] a) X. Zhang , D. Wang , Y. Han , F. Duan , Q. Lv , Z. Li , J. Assist. Reprod. Genet. 2014, 31, 1511; 25172095 10.1007/s10815-014-0320-2 PMC4389936

