# Supplementary material for: SINE‐Associated LncRNA SAWPA Regulates Porcine Zygotic Genome Activation
Source: Adv Sci (Weinh). 2023 Nov 20;11(2):2307505. doi: 10.1002/advs.202307505 (PMC10787077; doi:10.1002/advs.202307505)
Supplement: Supplementary file 1 — Supporting Information [file ADVS-11-2307505-s001.pdf]

ADVANCED SCIENCE
Open Access

Supporting Information

for *Adv. Sci.*, DOI 10.1002/advs.202307505

SINE-Associated LncRNA *SAWPA* Regulates Porcine Zygotic Genome Activation

Tianyao He, Jinyu Peng, Shu Yang, Dongsong Liu, Shuang Gao, Yanlong Zhu, Zhuang Chai, Byeong Chun Lee, Renyue Wei, Jiaqiang Wang, Zhonghua Liu* and Jun-Xue Jin**

Supporting Information

SINE-Associated LncRNA *SAWPA* Regulates Porcine Zygotic Genome Activation

Tianyao He, Jinyu Peng, Shu Yang, Dongsong Liu, Shuang Gao, Yanlong Zhu, Zhuang Chai, Byeong Chun Lee, Renyue Wei, Jiaqiang Wang*, Zhonghua Liu*, Jun-Xue Jin*

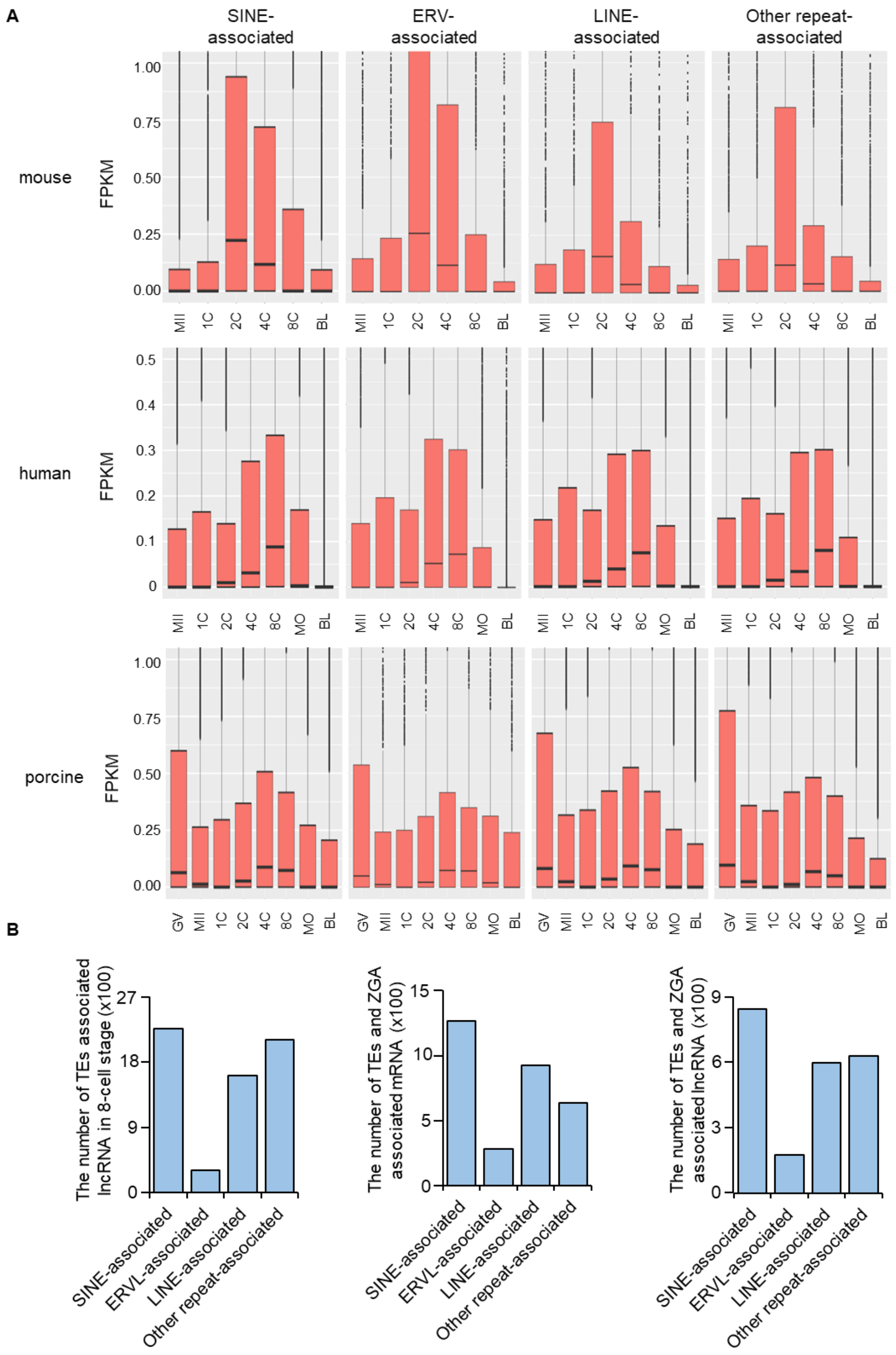

Figure. S1 The distribution patterns of TEs-associated lncRNA during early embryonic development in pigs, mice, and humans. A) The box plot for the expression levels analysis of TEs-associated lncRNA during early embryonic development in mouse, human and porcine (GSE138760, GSE36552, CRA004237). GV, germinal vesicle oocyst; MII, metaphase of second meiosis; 2C, 2-cell stage; 4C, 4-cell stage; 8C, 8-cell stage; MO, morula; BL, seven days blastocyst. B) The distribution of different types of TEs during the ZGA stage in porcine (CRA004237).

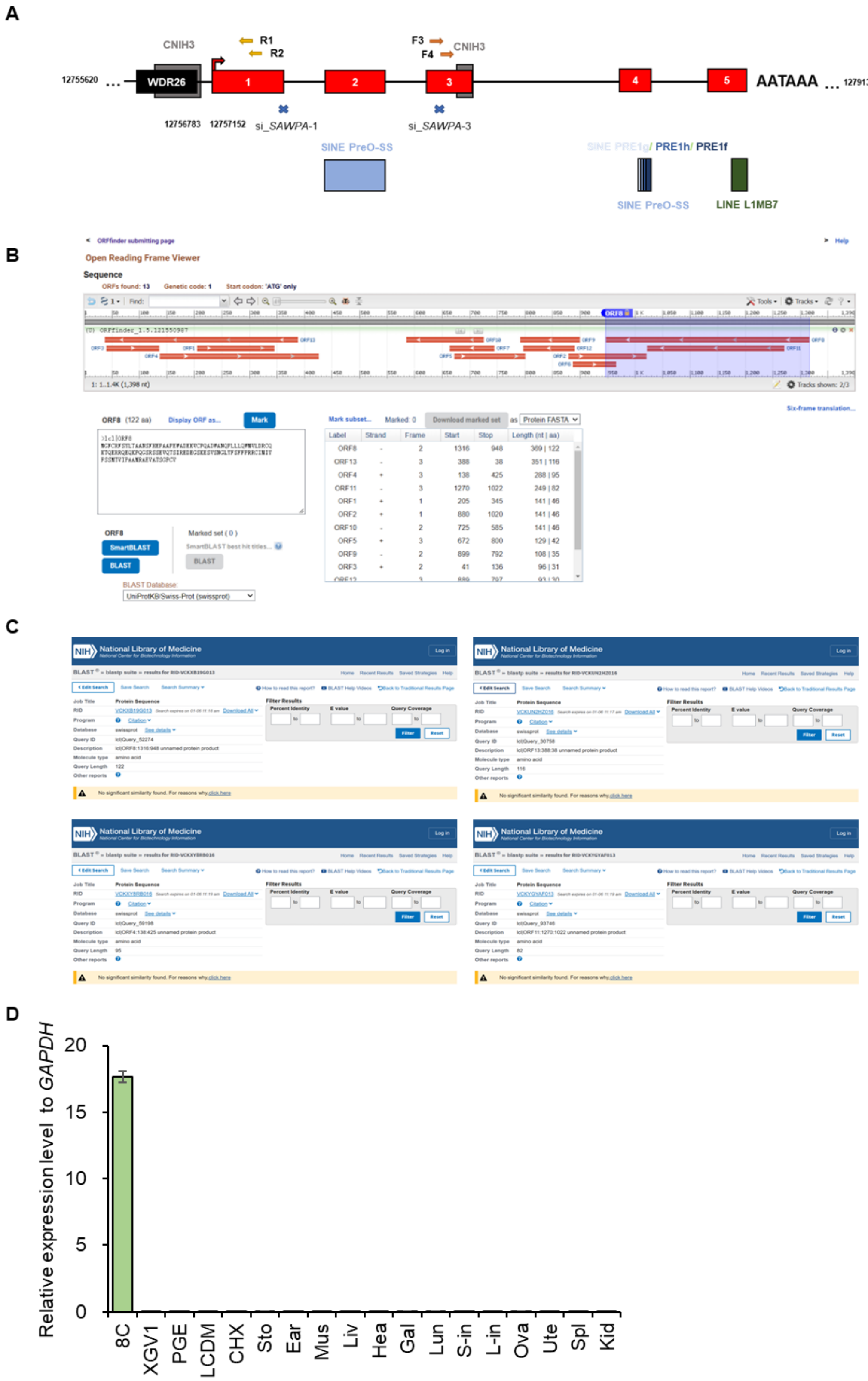

Figure. S2 *SAWPA* locus information and prediction of coding capacity. A) Gene locus of *SAWPA* RACE primers, RT-PCR primers and interference sites. AATAAA is the polyadenylated signal site. B) ORF analysis of *SAWPA*. C) Computational secondary structure analysis of *SAWPA*. D) *SAWPA* are undetectable in various tissues, porcine ESCs. 8-cell, 8-cell stage. Three experimental replicates were used, and the error bars represent s.e.m.

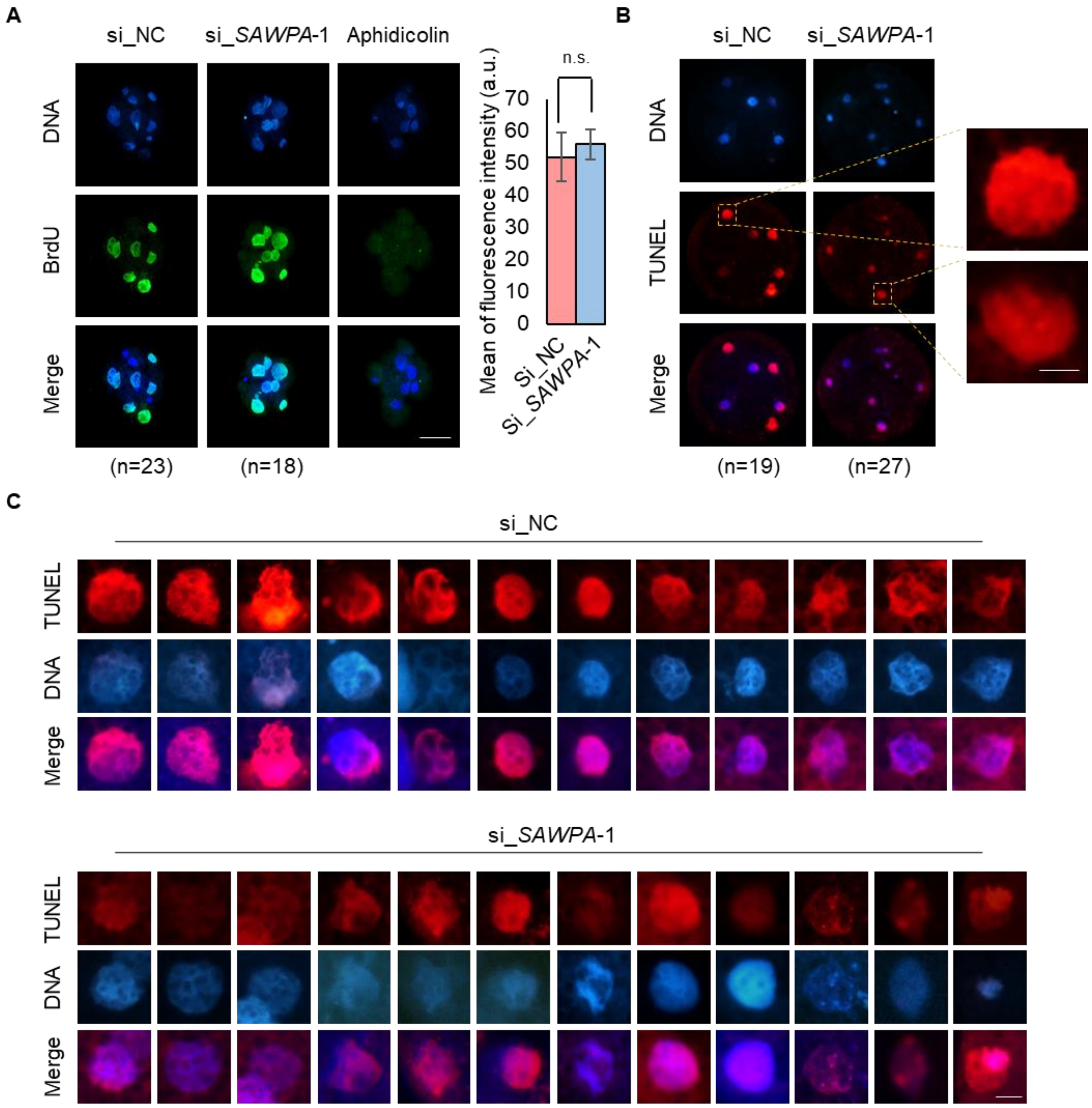

Figure. S3. si_*SAWPA*-1 embryos at 8-cell affected chromatin accessibility. A) si_*SAWPA*-1 treatment results in developmental arrest at 8-cell stage without affecting DNA integrity and replication. We used BrdU to visualize S and G2 phases. Aphidicolin-treated embryos were arrested at the S phase without DNA replication. There is no difference in si_NC and si_*SAWPA*-1 fluorescence intensity. The embryos were collected at pPA 72 h at the 8-cell stage for IF analysis. Scale bar, 50 μm. Three experimental replicates were used. The error bars represent S.E.M. B) *SAWPA* depletion led to significantly lower levels of TUNEL fluorescence. Scale bar, 10 μm. Three experimental replicates were used. C) DNase I TUNEL Fluorescence of the nucleus in the si_*SAWPA*-1 group and the si_NC group. Scale bar, 10 μm. Three experimental replicates were used.

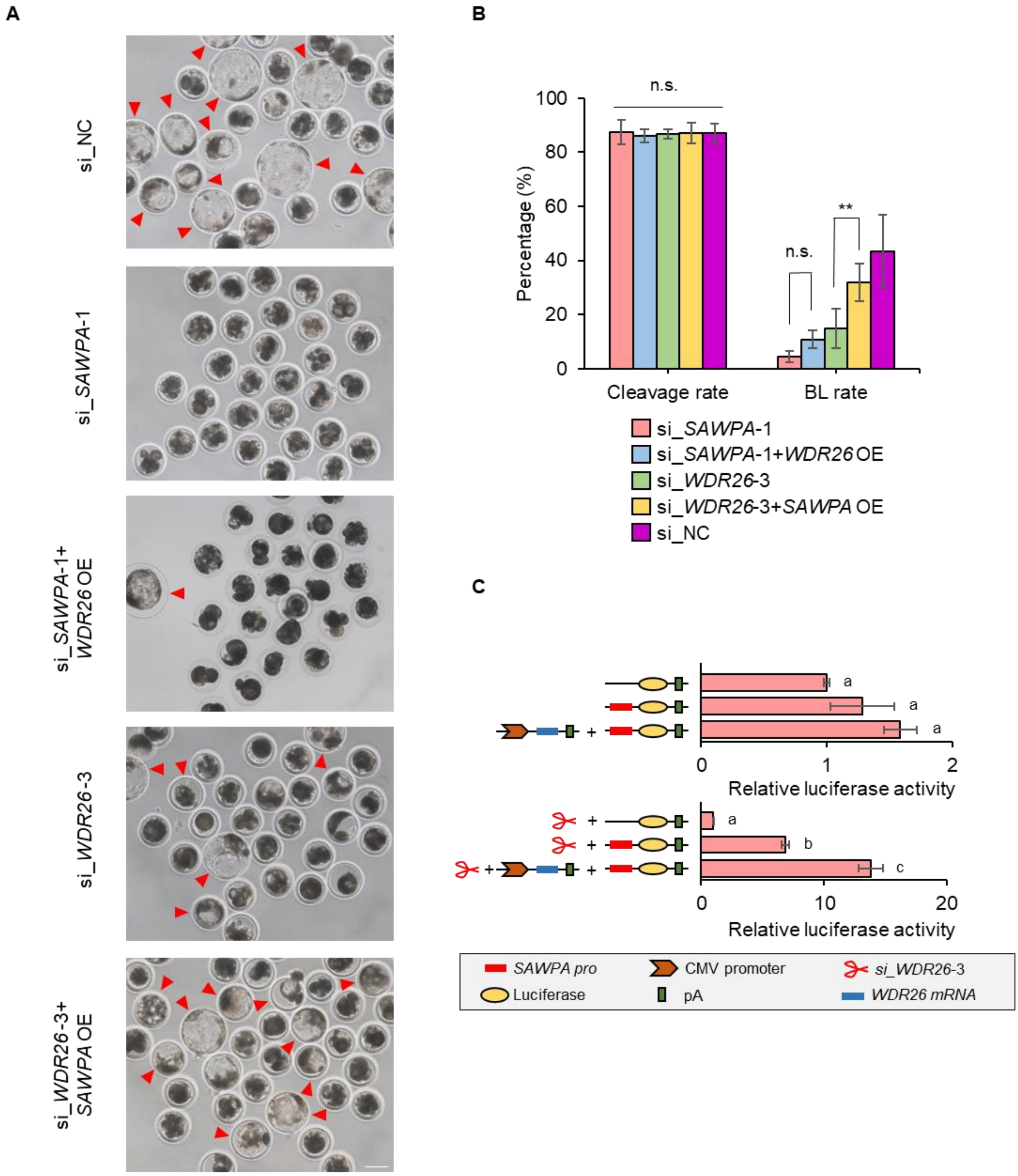

Figure. S4. *SAWPA* is located downstream of *WDR26*. A) Photographs of the si_*SAWPA*-1, *si_WDR26-3*, *si_WDR26-3* + *SAWPA OE*, and si_*SAWPA*-1 + *WDR26* OE injected embryo. The photographs were taken at pPA 168 h at the blastocyst stage. Embryos injected with si_NC and *si_WDR26-3* + *SAWPA OE* can develop to the blastocyst stage, while si_*SAWPA*-1, *si_WDR26-3*, *and* si_*SAWPA*-1 + *WDR26* OE embryos are arrested at the 8-cell stage. Scale bar, 100 μm. At least three experimental replicates were used for each RNAi injection (**Figure 3**D). B) Cleavage rate and blastocyst rate of the si_*SAWPA*-1, *si_WDR26-3*, *si_WDR26-3* + *SAWPA* OE, and si_*SAWPA*-1 + *WDR26* OE injected embryo. There was no significant

difference in cleavage rate, but *si_WDR26-3* + *SAWPA* significantly rescued *si_WDR26-3* blastocyst rate. Three experimental replicates were used. Two-tailed Student's *t*-test was used for statistical analysis, and the error bars represent S.E.M. C) Enhancer model analyses with the *SAWPA* promoter. The promoter DNA sequence of *SAWPA* was placed at the front of the firefly luciferase gene and PK15 cells were co-transfected with *WDR26* overexpression vector and Renilla luciferase reporter to observe fluorescence intensity. *WDR26* can enhance the activity of the *SAWPA* promoter in the case of interfering with endogenous *WDR26*. Three experimental replicates were used. Two-tailed Student's *t*-test was used for statistical analysis, and the error bars represent S.E.M.

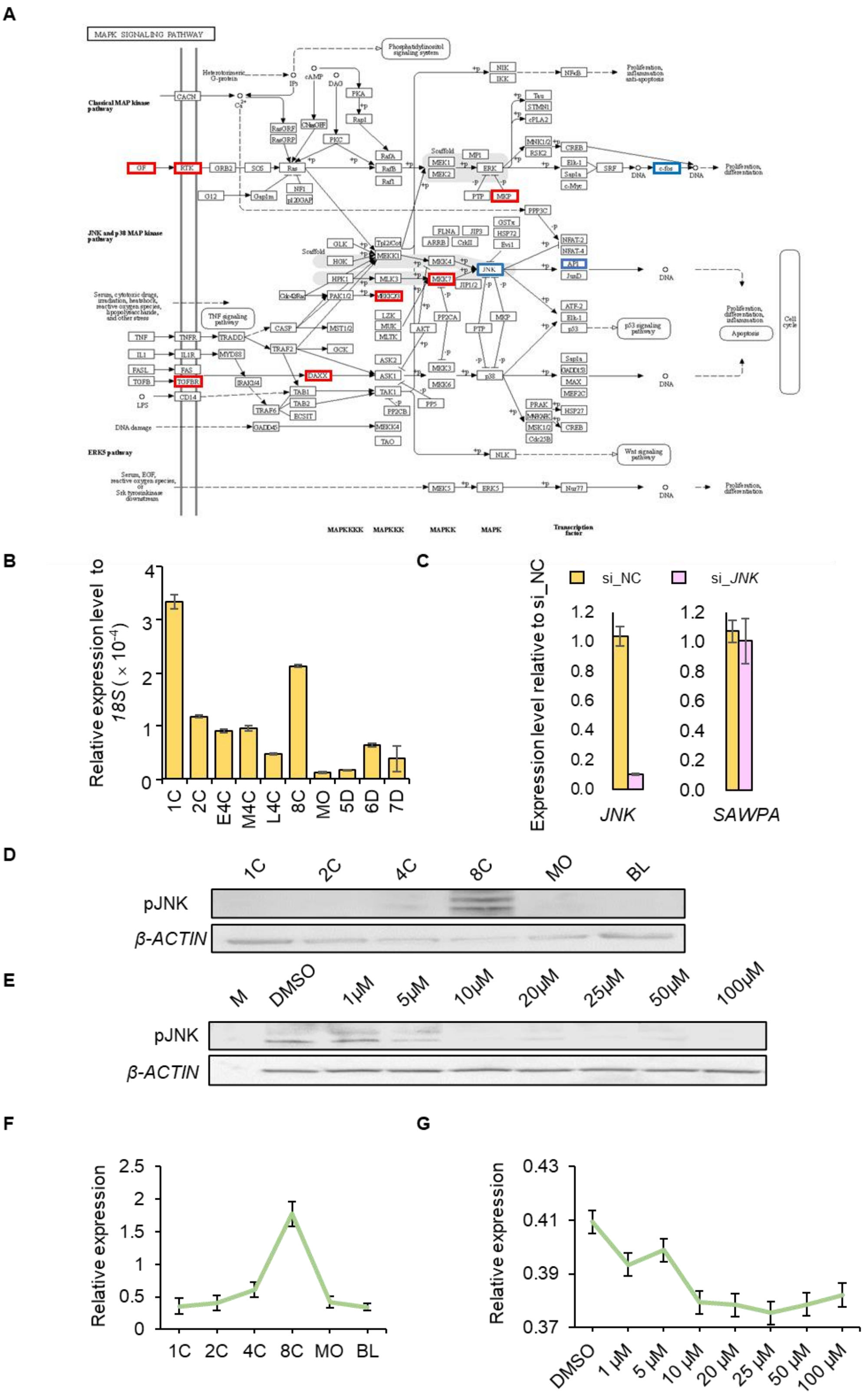

Figure. S5. JNK signaling pathway is important for ZGA of preimplantation porcine embryos. A) *SAWPA* depletion results in the inhibition of the MAPK signaling pathway. Upregulated genes are shown in red, while downregulated genes are shown in blue. B) Expression pattern of *JNK* in preimplantation embryos measured using qPCR. GV, germinal

vesicle oocyst; 1C, one cell stage; 2C, 2-cell stage; E4C, early 4-cell stage; M4C, middle 4-cell stage; L4C, late 4-cell stage; 8C, 8-cell stage; MO, morula; 5D, five days blastocyst. 6D, six days blastocyst; 5D, seven days blastocyst. The error bars represent S.E.M. Approximately 50 embryos of each stage were used, and three experimental replicates were used. C) *SAWPA* and *JNK* expression analysis relative to the control group after RNA interference (RNAi). si_NC, RNAi of negative control; si_*JNK*, RNAi of *JNK*. The error bars represent S.E.M. Approximately 50 embryos of each stage were used, and three experimental replicates were used. D) The expression of pJNK at various stages of embryonic development was detected using western blotting. pJNK, phosphorylated JNK. Approximately 200 embryos were used for each lane. Three experimental replicates were used. E) Western blotting was used to detect pJNK expression at the 8-cell stage after treatment of embryos at different TCS *JNK* 6o concentrations. About 200 embryos were used for each lane. Three experimental replicates were used.Type or paste caption here. Create a page break and paste in the Table above the caption. F) Western blot grayscale analysis indicate that the protein levels of pJNK, key kinases in the MAPK signaling pathway, increased in 8-cell stage. About 300 embryos were used for each lane. The error bars represent S.E.M. Three experimental replicates were used. G) Western blot grayscale analysis indicate that protein levels of pJNK were not influence after the 1μM and 5 μM pJNK inhibition treatment. About 200 embryos were used for each lane. The error bars represent S.E.M. Three experimental replicates were used.

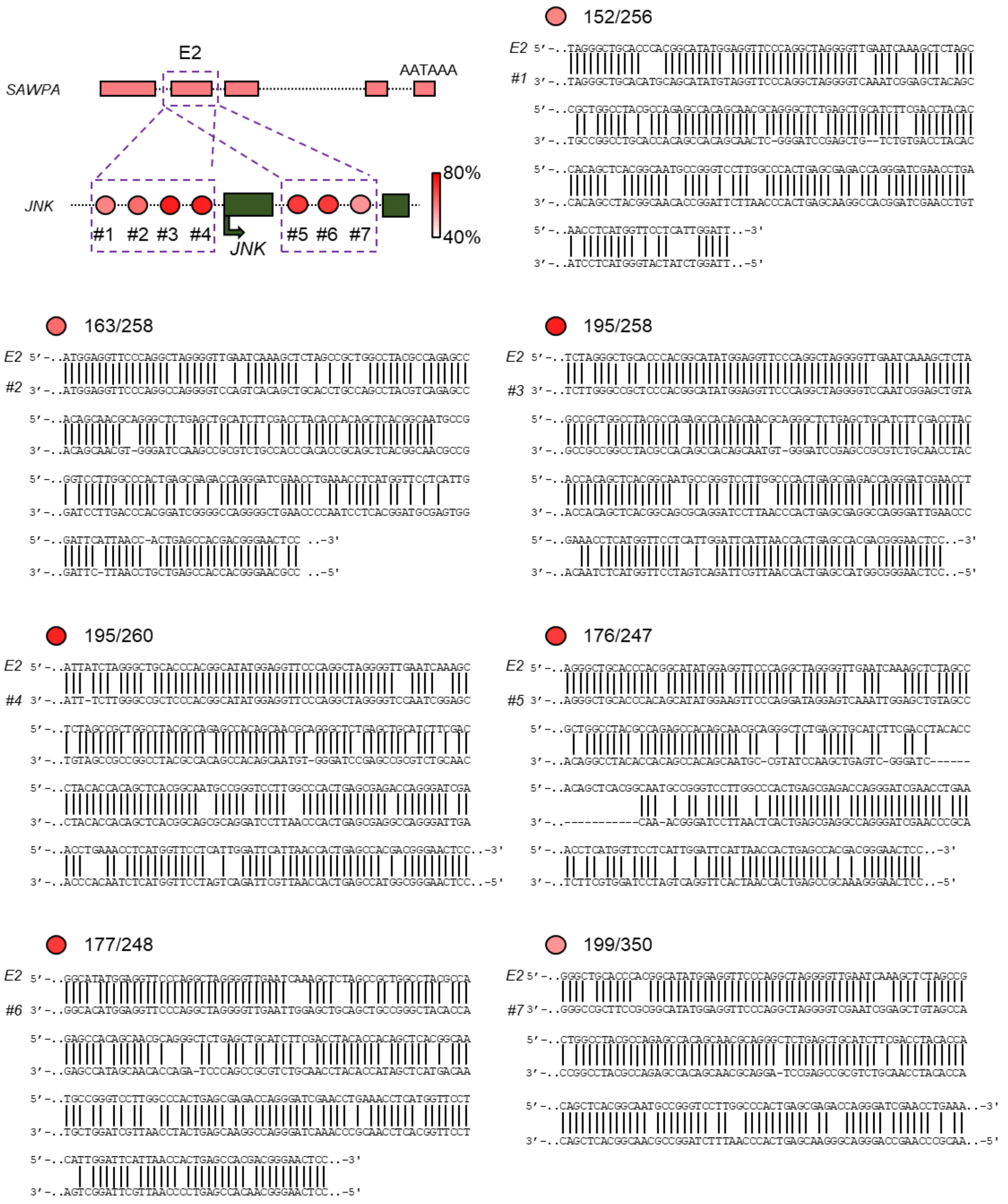

Figure. S6. The SINE sequences of *SAWPA* and *JNK* have different base matching information. Specific information on the complementary pairing of *SAWPA* exons 2 with 7 SINE sequence bases of *JNK*.

Table S1. Sequence of primers and FISH probe

Gene		Sequence (5'-3')	Length (bp)
SAWPA-1	forward	AGTTGCCTCTTGAGTCACTGCCC	124
	reverse	GCCTCCTATTTCACCTAAACATTTCG	
SAWPA-2	forward	AATTGGGTGCTGCCTCTGAAGAT	79
	reverse	GGCCGTGGTCCATGTGTGTACA	
WDR26	forward	CGTGCAAAAGCAGAATGGGAAG	94
	reverse	GAAGCATCACTGATGGTGGTAAATAGG	
TFIIA	forward	TCAGGAATAGAGTCAACTTCA	148
	reverse	TTACCATCACAGGCTACAA	
EIF3A	forward	TAACACAGACGACGACAG	197
	reverse	CATCATCCACACCTCCTC	
EIF1A	forward	GGTGTTCAAAGAAGATGGGCAAGAG	115
	reverse	TTTCCCTCTGATGTGACATAACCTC	
ACLY	forward	GGCCTTTCGTAGAGAGCAGG	145
	reverse	TGACCCGGGCATACTTGAAC	
SQLE	forward	AGGCGGTTATTCTGGTTACCG	129
	reverse	AACGGCAGTTTCCAGAGCG	
ACSS1	forward	GGTGAAGAAGTACGACCGCT	146
	reverse	CACCCGTTTCTGTCTGCCA	
PDHA1	forward	GGATTGCTCTGGCCTGTAAG	181
	reverse	GCTCTCTCCACAGACGTTCC	
Hsp70	forward	AGGACAGGTGCAAAGTACAAGACAA	158
	reverse	AGGGATACCAGGAGCCCCATAG	
ZSCAN4	forward	AGGTAAAGAATGTGGCGGCAAC	91
	reverse	GAGCAGGAAGGGCGTCTGACT	
ASH2L	forward	CTGACTGTGGTTGGAGAG	223
	reverse	CAATGGACTGGTGGAACT	
SMYD3	forward	TCTGCTACCTGGATATGC	191
	reverse	CCAATGTGCCTTCAGTTC	
Nid2	forward	TCCTACAGCATCCACCAGAACATC	107
	reverse	AAGGCAAAGACACGGTCCACAT	
Klf5	forward	GACGCGATGTGAAATGGAGAAGTA	176
	reverse	GGTGAGTGATGTCAGGGAGGAAGA	
OCT4	forward	TCAGCCAAACGACCATCT	96
	reverse	CTTCCTCCACCCACTTCT	
NANOG	forward	TCCTCTTCCTTCCTCCAT	108
	reverse	TCCTTCTCTGTGCTCTTC	
SOX2	forward	AACCAGAAGAACAGCCCAGAC	155
	reverse	TCCGACAAAAGTTTCCACTCG	
JNK	forward	AATGTTGCAATCAAGAAGTTAAGCC	188
	reverse	GCATCCATGAGCTCCATAACTATGT	
FOS	forward	CGGTGACTGCTATCTCGACCAG	157
	reverse	ATGGTCTTCACGACTCCAGCC	
FAS	forward	AAGATACAGCTGAACAGAAAGTCCAG	86
	reverse	TTGAATCAAAGTGCAATAGGCGT	
MAP2K7	forward	GCGACAGGACAGTTTCCCTACAAG	137
	reverse	AGGCAGTCTTTGACAAAGGACTGAA	
DUSP6	forward	CTGTGACTGTGGCTTATCTTATGCA	113
	reverse	CTGGCCCATGAAGTTGAAGTTG	

MAP3K2	forward	ATTGGATGAGCCCTGAAGTGATTAG	212
	reverse	CCGTTTGAGGAAATCTCGAGTATAGTC	
DAXX	forward	CTACAACTTCGGCTGTCACCTCAC	146
	reverse	TCATTGCATATTTGGAGATGACCTC	
FGF16	forward	GACTAAACGACACCAGAAATTCACTCA	70
	reverse	CATGGAGGGCAACTTAGAAGGAT	
FGFR1	forward	TCCTGTGGGAAATCTTCACTCTGG	263
	reverse	GTACTGGTCCAGGGGCATCGA	
PDGFC	forward	ACCAGGGTTCTGCATCCACTACAA	121
	reverse	AAAGGCAGTGACAGCATTGTTAAGC	
LTA	forward	GGACTCACTGCGCTGGAGAGC	111
	reverse	GAGTAGACAAAGTAGAGGCCACTGGTG	
ITGA5	forward	CCAAGCCCGAAGCAGTGATATT	114
	reverse	CCAGGTTGATGAGCTCGTAGACG	
ITGA7	forward	GTTTGGTGCGAGCAGAGGAGC	77
	reverse	CAGAATGACCACAGCACCCTTG	
ITGB3	forward	ACCGTTACTGTCGTGATGAGATTGA	151
	reverse	ACCACATACAGGATGGACTTTCCAC	
LPAR3	forward	AGTGACCTTCTTCATCATGGTTGTG	146
	reverse	AGACAGCCATCACCGTCTTCATTAG	
COL4A1	forward	ACAAATGGAATTGTGGAATGCCA	95
	reverse	TCTCCAACTTCGCCCGTCAAC	
GNG4	forward	TGTATGGACAGGGTGAAGGTGTC	133
	reverse	AGAAGAACTTCTTCTCTCGGAAAGG	
HSP90B1	forward	CCGTGAGACTCTGCAGCAACATA	89
	reverse	CAGCAATCTTCTTGATCATGTCCAG	
ITGB4	forward	CTACCTGGTGACGTGTGAGATGGC	127
	reverse	TGGCCTGCACCTTGAACTTGTAG	
18s	forward	TCCAATGGATCCTCGCGGAA	149
	reverse	GGCTACCACATCCAAGGAAG	
U6	forward	GCTCACTTCAGCAGCACATATAC	83
	reverse	CTTTACGAATTTTCATGGCATC	
GAPDH	forward	TCCTGGAAGATGGTGATGGCCTTT	160
	reverse	GCAAAGTGGACATTGTCGCCATCA	
FISH-*SAWPA*-1	forward	GTCTACTCTTCCAGACAGGGGTCCGACTAACCCG	34
	reverse	CGGGTTAGTCGGACCCCTGTCTGGAAGAGTAGAC	
FISH-*SAWPA*-2	forward	GCGTTCGTAAACCCGGCACCTTTAAGGTCAAAAG	34
	reverse	CTTTTGACCTTAAAGGTGCCGGGTTTACGAACGC	
SINE(3)	forward	GCATATGGAGGTTCCCAGGCTA	104
	reverse	GTGTAGGTCGAAGATGCAGCTCAGA	
SINE-JNK	forward	TGGTGTAGGTTGCAGATGCGG	82
	reverse	GCTAGGGGTTGAATCAGAGCTACAG	

Table S2. Correlation P-values for difference analysis of TEs-associated lncRNA

Porcine SINE associated lncRNA							
p values	GV	MII	2C	4C	8C	MO	BL
GV							
MII	2.59E-06						
2C	5.36E-03	2.11E-02					
4C	3.42E-01	2.01E-04	3.86E-02				
8C	3.93E-01	1.85E-04	3.33E-02	4.57E-01			
MO	4.78E-13	2.49E-02	1.09E-05	1.07E-08	1.35E-08		
BL	4.27E-16	3.18E-03	1.97E-07	1.10E-10	1.66E-10	2.08E-01	

Porcine ERV associated lncRNA							
p values	GV	MII	2C	4C	8C	MO	BL
GV							
MII	2.08E-02						
2C	7.07E-02	1.97E-01					
4C	3.76E-01	5.19E-02	1.47E-01				
8C	4.23E-01	5.63E-02	1.45E-01	4.61E-01			
MO	5.08E-04	9.29E-02	6.02E-03	2.63E-03	4.54E-03		
BL	2.38E-04	5.93E-02	2.39E-03	1.42E-03	2.75E-03	3.98E-01	

Porcine LINE associated lncRNA							
p values	GV	MII	2C	4C	8C	MO	BL
GV							
MII	7.63E-05						
2C	3.85E-03	3.83E-02					
4C	6.27E-02	2.44E-03	9.03E-02				
8C	6.24E-02	1.05E-03	6.38E-02	4.72E-01			
MO	3.68E-05	1.90E-01	1.20E-02	1.02E-03	5.43E-04		
BL	1.77E-05	1.28E-01	6.36E-03	4.99E-04	2.56E-04	4.07E-01	

Porcine Other repeat associated lncRNA							
p values	GV	MII	2C	4C	8C	MO	BL
GV							
MII	1.64E-05						
2C	1.01E-03	8.69E-02					
4C	5.05E-02	2.56E-03	5.90E-02				
8C	4.29E-02	2.73E-03	6.41E-02	4.74E-01			
MO	9.04E-06	2.67E-01	3.58E-02	1.13E-03	1.20E-03		
BL	1.03E-06	9.32E-02	7.30E-03	1.57E-04	1.66E-04	2.53E-01	

Mouse SINE associated lncRNA						
p values	GV	1C	2C	4C	8C	BL
GV						
1C	6.07E-02					
2C	2.31E-02	2.80E-01				
4C	3.32E-05	2.96E-04	9.50E-04			
8C	4.47E-02	4.80E-01	2.57E-01	5.09E-05		
BL	3.69E-01	8.87E-03	2.26E-04	1.00E-10	3.33E-04	

Human ERV associated lncRNA							
p values	MII	1C	2C	4C	8C	MO	BL
MII							
1C	2.71E-01						
2C	1.77E-02	5.03E-02					
4C	5.40E-02	1.41E-01	2.57E-01				
8C	8.67E-18	8.31E-20	2.70E-11	7.70E-17			
MO	2.01E-04	6.60E-04	9.78E-02	1.55E-02	1.79E-11		
BL	8.64E-36	1.23E-42	2.32E-28	3.04E-40	8.56E-26	3.96E-36	

Table S3. Correlation P-values for difference analysis of Embryonic development after microinjection

BL rates												
p values	si_NC	si_*SAWPA*-1	si_*SAWPA*-2	si_*SAWPA*-3	si_*SAWPA*-4	si_*WDR26*-1	si_*WDR26*-2	si_*WDR26*-3	si_*WDR26*-3+*SAWPA* OE	si_*SAWPA*-1+*WDR26* OE	*SAWPA* OE	si_*CNIH3*
si_NC												
si_*SAWPA*-1	1.71E-04											
si_*SAWPA*-2	3.32E-03	8.87E-04										
si_*SAWPA*-3	1.62E-03	4.97E-02	2.69E-02									
si_*SAWPA*-4	2.09E-03	1.43E-03	2.65E-02	1.43E-01								
si_*WDR26*-1	1.09E-01	2.29E-03	3.46E-02	1.53E-02	2.07E-02							
si_*WDR26*-2	3.21E-02	2.29E-03	5.79E-02	1.96E-02	2.87E-02	2.50E-01						
si_*WDR26*-3	4.21E-03	1.15E-02	3.97E-01	1.27E-01	2.70E-01	4.03E-02	7.09E-02					
si_*WDR26*-3+*SAWPA* OE	2.37E-02	6.61E-05	4.98E-03	1.25E-03	1.96E-03	3.83E-01	2.29E-01	7.80E-03				
si_*SAWPA*-1+*WDR26* OE	4.62E-04	8.11E-03	1.91E-02	3.17E-01	2.02E-01	6.82E-03	9.96E-03	1.34E-01	4.24E-04			
SAWPA OE	1.56E-01	9.55E-08	3.54E-06	2.26E-05	3.46E-06	1.73E-01	1.99E-02	3.26E-04	2.73E-02	1.94E-06		
si_*CNIH3*	8.33E-05	2.61E-02	2.84E-03	3.87E-01	3.96E-02	1.77E-03	2.48E-03	4.18E-02	6.25E-05	1.62E-01	2.18E-01	

2C arrest rates												
p values	si_NC	si_*SAWPA*-1	si_*SAWPA*-2	si_*SAWPA*-3	si_*SAWPA*-4	si_*WDR26*-1	si_*WDR26*-2	si_*WDR26*-3	si_*WDR26*-3+*SAWPA* OE	si_*SAWPA*-1+*WDR26* OE	*SAWPA* OE	si_*CNIH3*
si_NC												
si_*SAWPA*-1	1.21E-01											
si_*SAWPA*-2	1.24E-01	3.54E-01										
si_*SAWPA*-3	3.52E-02	1.72E-01	1.41E-01									
si_*SAWPA*-4	3.67E-02	3.69E-01	2.17E-01	1.99E-01								
si_*WDR26*-1	2.49E-01	5.79E-02	3.16E-02	2.36E-02	1.48E-03							
si_*WDR26*-2	1.30E-02	1.76E-01	6.14E-02	3.68E-01	3.09E-03	6.34E-04						
si_*WDR26*-3	4.22E-02	4.03E-01	3.40E-01	2.51E-01	4.92E-01	4.55E-02	2.60E-01					
si_*WDR26*-3+*SAWPA* OE	7.35E-02	3.74E-01	3.22E-01	2.37E-01	4.85E-01	3.95E-02	2.92E-01	4.82E-01				
si_*SAWPA*-1+*WDR26* OE	2.86E-01	2.88E-01	3.30E-01	9.42E-02	1.76E-01	1.51E-01	7.86E-02	2.32E-01	1.93E-01			
SAWPA OE	2.84E-01	5.01E-02	3.60E-02	1.52E-02	4.55E-03	4.24E-01	1.55E-03	3.77E-02	3.05E-02	1.49E-01		
si_*CNIH3*	5.03E-05	8.54E-05	3.44E-04	6.20E-04	3.76E-04	2.07E-04	4.55E-04	4.31E-04	2.43E-05	6.97E-05	2.67E-01	

8C arrest rates												
p values	si_NC	si_*SAWPA*-1	si_*SAWPA*-2	si_*SAWPA*-3	si_*SAWPA*-4	si_*WDR26*-1	si_*WDR26*-2	si_*WDR26*-3	si_*WDR26*-3+*SAWPA* OE	si_*SAWPA*-1+*WDR26* OE	*SAWPA* OE	si_*CNIH3*
si_NC												
si_*SAWPA*-1	6.93E-05											
si_*SAWPA*-2	1.11E-02	3.04E-04										
si_*SAWPA*-3	6.86E-04	3.86E-02	1.87E-03									
si_*SAWPA*-4	8.32E-04	1.70E-01	4.64E-03	3.89E-01								
si_*WDR26*-1	6.66E-02	1.02E-02	3.86E-01	4.16E-02	4.07E-02							
si_*WDR26*-2	3.17E-01	1.86E-04	2.42E-02	1.75E-03	2.35E-03	1.35E-01						
si_*WDR26*-3	1.64E-02	7.29E-03	3.44E-01	3.96E-02	4.14E-05	3.13E-01	4.01E-02					
si_*WDR26*-3+*SAWPA* OE	3.27E-01	8.68E-07	1.25E-03	1.99E-05	4.14E-05	4.52E-02	3.99E-01	5.02E-03				
si_*SAWPA*-1+*WDR26* OE	9.11E-04	7.51E-03	2.51E-02	7.38E-02	7.18E-02	7.94E-02	2.74E-03	1.31E-01	7.44E-05			
SAWPA OE	4.64E-01	2.23E-05	5.90E-03	2.84E-04	4.04E-04	6.11E-02	3.24E-01	1.22E-02	3.47E-01	4.77E-04		
si_*CNIH3*	6.33E-04	1.61E-11	1.60E-07	4.41E-09	1.39E-07	5.08E-04	2.68E-04	3.28E-05	2.66E-06	1.64E-07	2.18E-01	

4-8C arrest rates												
p values	si_NC	si_*SAWPA*-1	si_*SAWPA*-2	si_*SAWPA*-3	si_*SAWPA*-4	si_*WDR26*-1	si_*WDR26*-2	si_*WDR26*-3	si_*WDR26*-3+*SAWPA* OE	si_*SAWPA*-1+*WDR26* OE	*SAWPA* OE	si_*CNIH3*
si_NC												
si_*SAWPA*-1	2.25E-04											
si_*SAWPA*-2	1.31E-02	1.48E-03										
si_*SAWPA*-3	1.10E-03	3.96E-01	6.51E-03									
si_*SAWPA*-4	1.44E-03	6.52E-02	6.59E-03	1.31E-01								
si_*WDR26*-1	7.43E-02	3.37E-03	1.82E-01	1.11E-02	1.58E-02							
si_*WDR26*-2	1.79E-01	3.53E-04	2.54E-02	1.74E-03	1.59E-03	2.09E-01						
si_*WDR26*-3	3.21E-02	4.16E-03	3.33E-01	1.32E-02	1.96E-02	3.16E-01	8.78E-02					
si_*WDR26*-3+*SAWPA* OE	1.67E-01	3.23E-05	8.58E-03	1.97E-04	2.45E-04	1.21E-01	4.10E-01	3.67E-02				
si_*SAWPA*-1+*WDR26* OE	1.58E-03	2.03E-03	6.93E-02	7.09E-03	1.15E-02	3.72E-02	2.40E-03	7.27E-02	5.00E-04			
SAWPA OE	1.46E-03	9.60E-08	3.37E-06	1.22E-06	4.25E-08	3.19E-04	1.02E-04	8.94E-05	1.93E-05	6.08E-08		
si_*CNIH3*	3.08E-03	1.65E-07	1.23E-05	1.89E-06	9.96E-07	3.54E-04	2.76E-04	1.01E-04	5.32E-05	4.41E-07	3.42E-01	

Table S4. Correlation P-values for difference analysis of Embryonic development after si_*SAWPA*-1 and si_*JNK* microinjection

BL rates				
p values	si_*SAWPA*-1	si_*JNK*	si_*SAWPA*-1+*JNK* mRNA	si_NC
si_*SAWPA*-1				
si_*JNK*	7.57E-04			
si_*SAWPA*-1+*JNK* mRNA	4.55E-05	2.69E-02		
si_NC	1.79E-04	1.30E-02	1.10E-03	

2C arrest rates				
p values	si_*SAWPA*-1	si_*JNK*	si_*SAWPA*-1+*JNK* mRNA	si_NC
si_*SAWPA*-1				
si_*JNK*	2.45E-01			
si_*SAWPA*-1+*JNK* mRNA	3.62E-01	2.04E-01		
si_NC	1.06E-01	2.86E-01	1.05E-01	

8C arrest rates				
p values	si_*SAWPA*-1	si_*JNK*	si_*SAWPA*-1+*JNK* mRNA	si_NC
si_*SAWPA*-1				
si_*JNK*	7.10E-03			
si_*SAWPA*-1+*JNK* mRNA	3.14E-03	5.13E-02		
si_NC	3.46E-04	3.44E-03	7.77E-02	

4-8C arrest rates				
p values	si_*SAWPA*-1	si_*JNK*	si_*SAWPA*-1+*JNK* mRNA	si_NC
si_*SAWPA*-1				
si_*JNK*	1.91E-03			
si_*SAWPA*-1+*JNK* mRNA	1.28E-03	1.22E-03		
si_NC	7.20E-04	1.36E-02	1.16E-03	

Table S5. Correlation P-values for difference analysis of Embryonic development after JNK inhibition treatment

BL rates								
p values	1μM TCS JNK 6o	5μM TCS JNK 6o	10μM TCS JNK 6o	20μM TCS JNK 6o	25μM TCS JNK 6o	50μM TCS JNK 6o	100μM TCS JNK 6o	DMSO
1μM TCS JNK 6o								
5μM TCS JNK 6o	2.03E-01							
10μM TCS JNK 6o	3.42E-04	3.69E-03						
20μM TCS JNK 6o	3.83E-04	3.23E-03	3.89E-01					
25μM TCS JNK 6o	6.23E-04	7.92E-03	2.21E-01	3.09E-01				
50μM TCS JNK 6o	1.03E-04	1.58E-03	3.45E-02	6.52E-02	7.50E-02			
100μM TCS JNK 6o	1.46E-04	1.13E-03	1.54E-02	2.83E-02	1.00E-01	2.53E-01		
DMSO	3.69E-01	3.20E-01	1.66E-03	1.71E-03	3.48E-03	7.93E-04	8.83E-04	

2C arrest rates								
p values	1μM TCS JNK 6o	5μM TCS JNK 6o	10μM TCS JNK 6o	20μM TCS JNK 6o	25μM TCS JNK 6o	50μM TCS JNK 6o	100μM TCS JNK 6o	DMSO
1μM TCS JNK 6o								
5μM TCS JNK 6o	3.95E-01							
10μM TCS JNK 6o	2.04E-01	3.92E-01						
20μM TCS JNK 6o	8.60E-02	1.10E-01	1.02E-01					
25μM TCS JNK 6o	8.01E-02	2.06E-01	8.83E-02	4.27E-01				
50μM TCS JNK 6o	6.94E-02	5.81E-02	3.94E-02	1.77E-01	2.63E-01			
100μM TCS JNK 6o	1.57E-01	1.89E-01	1.52E-01	4.66E-01	4.32E-01	3.32E-01		
DMSO	4.22E-01	3.30E-01	1.64E-01	5.84E-02	8.88E-02	4.65E-02	1.27E-01	

8C arrest rates								
p values	1μM TCS JNK 6o	5μM TCS JNK 6o	10μM TCS JNK 6o	20μM TCS JNK 6o	25μM TCS JNK 6o	50μM TCS JNK 6o	100μM TCS JNK 6o	DMSO
1μM TCS JNK 6o								
5μM TCS JNK 6o	3.56E-01							
10μM TCS JNK 6o	1.77E-03	6.87E-03						
20μM TCS JNK 6o	1.62E-04	1.16E-03	4.93E-01					
25μM TCS JNK 6o	4.13E-03	1.94E-02	4.98E-01	4.97E-01				
50μM TCS JNK 6o	2.08E-03	1.60E-02	4.64E-01	4.55E-01	4.65E-01			
100μM TCS JNK 6o	3.16E-05	3.83E-04	2.57E-02	3.99E-03	4.22E-02	5.71E-03		
DMSO	2.99E-01	2.29E-01	1.80E-03	1.45E-04	3.61E-03	1.31E-03	3.73E-05	

4-8C arrest rates								
p values	1μM TCS JNK 6o	5μM TCS JNK 6o	10μM TCS JNK 6o	20μM TCS JNK 6o	25μM TCS JNK 6o	50μM TCS JNK 6o	100μM TCS JNK 6o	DMSO
1μM TCS JNK 6o								
5μM TCS JNK 6o	4.92E-01							
10μM TCS JNK 6o	9.11E-03	1.46E-02						
20μM TCS JNK 6o	2.38E-03	4.71E-03	4.40E-01					
25μM TCS JNK 6o	9.48E-03	2.11E-02	3.63E-01	3.77E-01				
50μM TCS JNK 6o	2.40E-02	4.13E-02	4.62E-01	5.00E-01	4.14E-01			
100μM TCS JNK 6o	3.30E-04	1.07E-03	2.46E-02	1.02E-02	2.41E-02	6.53E-02		
DMSO	1.09E-01	1.37E-01	2.64E-03	5.24E-04	3.61E-03	1.01E-02	2.50E-04	

Table S6. Correlation P-values for difference analysis of overexpressed six RNA fragments upon *SAWPA* depletion

BL rates							
p values	si_*SAWPA*-1	si_*SAWPA*-1+Δ1	si_*SAWPA*-1+Δ2	si_*SAWPA*-1+Δ3	si_*SAWPA*-1+Δ4	si_*SAWPA*-1+Δ5	si_*SAWPA*-1+Mut
si_*SAWPA*-1							
si_*SAWPA*-1+Δ1	1.02E-05						
si_*SAWPA*-1+Δ2	8.00E-05	2.16E-01					
si_*SAWPA*-1+Δ3	3.93E-01	3.62E-07	4.79E-06				
si_*SAWPA*-1+Δ4	1.09E-08	2.02E-02	1.89E-01	6.17E-10			
si_*SAWPA*-1+Δ5	1.27E-06	4.27E-01	2.29E-01	3.52E-08	1.36E-02		
si_*SAWPA*-1+Mut	3.46E-05	2.98E-01	3.90E-01	1.43E-06	6.56E-02	3.29E-01	

2C arrest rates							
p values	si_*SAWPA*-1	si_*SAWPA*-1+Δ1	si_*SAWPA*-1+Δ2	si_*SAWPA*-1+Δ3	si_*SAWPA*-1+Δ4	si_*SAWPA*-1+Δ5	si_*SAWPA*-1+Mut
si_*SAWPA*-1							
si_*SAWPA*-1+Δ1	3.62E-01						
si_*SAWPA*-1+Δ2	6.45E-02	4.34E-02					
si_*SAWPA*-1+Δ3	6.48E-02	6.22E-03	5.39E-04				
si_*SAWPA*-1+Δ4	4.55E-01	2.89E-01	3.48E-02	5.84E-02			
si_*SAWPA*-1+Δ5	2.85E-02	1.26E-02	4.29E-01	5.38E-05	1.25E-02		
si_*SAWPA*-1+Mut	2.37E-01	8.00E-02	1.05E-02	2.11E-01	2.43E-01	2.37E-03	

8C arrest rates							
p values	si_*SAWPA*-1	si_*SAWPA*-1+Δ1	si_*SAWPA*-1+Δ2	si_*SAWPA*-1+Δ3	si_*SAWPA*-1+Δ4	si_*SAWPA*-1+Δ5	si_*SAWPA*-1+Mut
si_*SAWPA*-1							
si_*SAWPA*-1+Δ1	8.09E-06						
si_*SAWPA*-1+Δ2	8.06E-06	4.21E-01					
si_*SAWPA*-1+Δ3	6.77E-06	2.18E-05	2.35E-05				
si_*SAWPA*-1+Δ4	7.83E-07	3.97E-01	4.88E-01	1.79E-06			
si_*SAWPA*-1+Δ5	2.02E-09	4.45E-01	3.32E-01	3.06E-09	2.61E-01		
si_*SAWPA*-1+Mut	4.84E-07	3.43E-01	4.27E-01	5.82E-07	4.17E-01	1.35E-01	

4-8C arrest rates							
p values	si_*SAWPA*-1	si_*SAWPA*-1+Δ1	si_*SAWPA*-1+Δ2	si_*SAWPA*-1+Δ3	si_*SAWPA*-1+Δ4	si_*SAWPA*-1+Δ5	si_*SAWPA*-1+Mut
si_*SAWPA*-1							
si_*SAWPA*-1+Δ1	1.85E-05						
si_*SAWPA*-1+Δ2	3.51E-06	4.64E-01					
si_*SAWPA*-1+Δ3	5.27E-04	1.04E-04	2.20E-05				
si_*SAWPA*-1+Δ4	7.76E-04	1.85E-04	1.85E-04	6.37E-05			
si_*SAWPA*-1+Δ5	1.73E-04	3.42E-05	3.41E-05	1.00E-05	3.16E-01		
si_*SAWPA*-1+Mut	8.96E-04	1.99E-04	1.98E-04	6.53E-05	2.52E-01	1.20E-01	
